# Intensity-based analysis of dual-color gene expression data as an alternative to ratio-based analysis to enhance reproducibility

**DOI:** 10.1186/1471-2164-11-112

**Published:** 2010-02-17

**Authors:** Koen Bossers, Bauke Ylstra, Ruud H Brakenhoff, Serge J Smeets, Joost Verhaagen, Mark A van de Wiel

**Affiliations:** 1 Neuroregeneration Laboratory, Netherlands Institute for Neuroscience, Meibergdreef 47, 1105 BA Amsterdam, the Netherlands; 2 Microarray Core Facility, Department of Pathology, VU University Medical Center, PO Box 7075, 1007 MB Amsterdam, the Netherlands; 3 Section of Tumour Biology, Department of Otolaryngology/Head-Neck Surgery, VU University Medical Center, De Boelelaan 1117, 1081 HV Amsterdam, the Netherlands; 4 Department of Mathematics, Vrije Universiteit, De Boelelaan 1081a, 1081 HV Amsterdam, the Netherlands; 5 Department of Biostatistics, VU University Medical Center, PO Box 7075, 1007 MB Amsterdam, the Netherlands

## Abstract

**Background:**

Ratio-based analysis is the current standard for the analysis of dual-color microarray data. Indeed, this method provides a powerful means to account for potential technical variations such as differences in background signal, spot size and spot concentration. However, current high density dual-color array platforms are of very high quality, and inter-array variance has become much less pronounced. We therefore raised the question whether it is feasible to use an intensity-based analysis rather than ratio-based analysis of dual-color microarray datasets. Furthermore, we compared performance of both ratio- and intensity-based analyses in terms of reproducibility and sensitivity for differential gene expression.

**Results:**

By analyzing three distinct and technically replicated datasets with either ratio- or intensity-based models, we determined that, when applied to the same dataset, intensity-based analysis of dual-color gene expression experiments yields 1) more reproducible results, and 2) is more sensitive in the detection of differentially expressed genes. These effects were most pronounced in experiments with large biological variation and complex hybridization designs. Furthermore, a power analysis revealed that for direct two-group comparisons above a certain sample size, ratio-based models have higher power, although the difference with intensity-based models is very small.

**Conclusions:**

Intensity-based analysis of dual-color datasets results in more reproducible results and increased sensitivity in the detection of differential gene expression than the analysis of the same dataset with ratio-based analysis. Complex dual-color setups such as interwoven loop designs benefit most from ignoring the array factor. The applicability of our approach to array platforms other than dual-color needs to be further investigated.

## Background

During the last decade, microarray technology has evolved into an indispensable tool for high-throughput gene expression studies. For example, microarrays are now routinely applied to identify differentially expressed genes between paired sample series, classify tumors in prognostic groups, and identify transcriptional alterations during development [1-3]. Two main types of commercial high density microarray platforms have emerged: one-color oligonucleotide platforms such as Affymetrix and Illumina, and dual-color oligonucleotide platforms such as Agilent and Nimblegen. Dual-color gene expression platforms are very efficient in directly comparing two conditions, by hybridizing the two conditions together on the same array. This greatly reduces the possible confounding effects of inter-array variability and local array effects.

The outcome of comparative microarray experiments is a ranked list of significant genes, possibly involved in the process under investigation. The resulting gene list then serves as a starting point for further investigations, such as constructing new hypotheses, or the *in vitro *characterization of putatively identified genes. Due to their dimensionality (few observations, many variables), microarray experiments suffer from high rates of false positive and negative findings [4]. Thus, the issue of reproducibility is of utmost importance in array experiments.

Analysis of variance (ANOVA) is a widely used tool to analyze and rank genes in both one- and dual-color comparative gene expression experiments [5]. The ANOVA model incorporates factors such as treatment, tissue and age to estimate the effect of interest per gene. For dual-color arrays specifically, an array effect is included in the model to determine the technical noise introduced by any between-array differences. Accounting for such an array effect in the analysis of dual-color arrays was initially necessary due to the relatively poor quality of array platforms: researchers were confronted with different levels of background signals across arrays, and the process of spotting cDNAs yielded probes with different shapes and probe concentrations. The latest generations of commercial dual-color platforms however use synthesized oligonucleotide probes instead of cDNA probes, and are of much higher and consistent quality and concentration. Subsequently, the variance introduced by the array effect has become much less pronounced [6]. We recently reported that using the Agilent arrayCGH platform or other CGH array platforms, the separate channels of these dual channel arrays are interchangeable, avoiding redundant hybridizations of the same reference material in every experiment [7]. We therefore raised the question whether the results obtained by separately analyzing intensities from co-hybridized gene expression array samples are more reproducible than results based on classical ratio-based analysis. As an added benefit, an intensity-based analysis approach allows for pairwise comparison between any samples.

We have performed a set of experiments to determine whether the intensity-based analysis of dual-color arrays is more reproducible than the conventional ratio-based analysis. Two independent datasets were used: a human keratinocyte cell line dataset, and a dataset based on human brain tissue. By selecting these datasets, we were able to study the performance of the ratio- and intensity-based models in two distinct situations: no biological variation (cell line dataset) versus substantial biological variation (brain dataset).

For both the intensity-based and ratio-based analysis, we estimated the reproducibility and sensitivity in the detection of differential gene expression by analyzing technical replicates. Technical replicates, consisting of two non-overlapping sets of microarrays, were used rather than biological replicates, as our focus is on inclusion or exclusion of the array factor, which is a technical factor. Furthermore, we used a model selection algorithm to determine whether either intensity or ratio based analysis was most suitable for each dataset.

Our results indicate that intensity-based analysis outperforms the standard ratio-based analysis of the same dataset. Intensity-based results are more reproducible, and increase the sensitivity of detecting regulated genes. Our results also indicate that differences between ratio- and intensity-based results become smaller in large datasets with simple designs, suggesting that more complex designs such as factorial and loop designs benefit most from our approach.

## Results

### Intensity-based analysis yields comparable results to ratio-based analysis, but is more sensitive in detecting differential gene expression

To determine the feasibility of intensity-based analysis of dual-color arrays, we performed a microarray experiment in which the effects of 4 different treatments (T1, T2, T3 and T4) were investigated using two keratinocyte-derived cell lines by measuring transcript levels on Agilent 4 × 44K Whole Human Genome arrays. The entire experiment was technically replicated (experiment C1 and C2, the hybridization setup can be found in Additional file 1).

**Figure 1:**
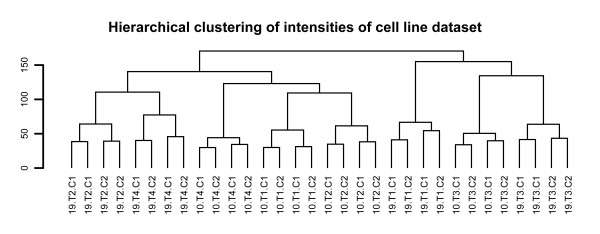
**Intensities of the same sample measured on separate arrays are highly correlated**. Hierarchical clustering of log2-transformed single channel intensities of the complete cell line experiment. Only genes with an average intensity A > 7 were used. Note that identical cell line-treatment combinations always cluster together, regardless of the co-hybridized sample. Sample naming = [cell line] [treatment] [duplicate set].

**Figure 2:**
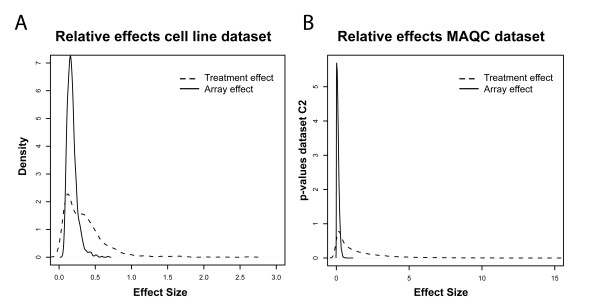
**Comparison of effect sizes for array and treatment factors**. Comparison between the relative sizes of the array and treatment effects, derived from the ANOVA model. Panel A: cell line dataset. Panel B: MAQC dataset. Dashed line: smoothed histogram over all genes for treatment effect size (absolute value of M-value), averaged over all treatment comparisons. Solid line: smoothed histogram over all genes for average array effect size (absolute value of M-value). Note that both the treatment and array effects still include an unavoidable noise component, hence one expects a partial overlap in the histograms because of genes that do not show a differential effect between treatments. Still there is a clear proportion of genes for which the mean treatment effect is much larger than the array effect size.

One of the prerequisites of intensity-based analysis of dual-color arrays is that co-hybridized samples do not influence gene expression measurements in the opposite channels. In other words, the intensity distribution of sample X should be independent of the co-hybridized sample. A hierarchical cluster analysis of the individual intensities of all arrays showed that cell line-treatment combinations invariably clustered together (Figure 1). This indeed suggests that intensities do not seem to be influenced by the co-hybridized sample or array used, as the array effect appears to be smaller than the treatment effect. To further investigate the magnitude of array-specific effects we compared the relative effect sizes of the array and treatment effects as defined by the ANOVA model. This analysis revealed that, apart from the noise component introduced by genes that are not differentially expressed between treatments, the treatment effect is much larger than the array effect (Figure 2A). Consequently, we expected the *in silico *reconstructed ratios between two samples that were not co-hybridized, to be very similar to the directly measured ratios between those samples. We indeed observed a strong linear correlation between the directly measured ratios, and the *in silico *reconstructed ratios based on separate hybridizations (average correlation 0.78, range 0.47-0.88, Additional file 2).

To investigate whether these findings translate to other datasets as well, we analyzed a publicly available dataset in which two commercially available samples were hybridized 10 times on Agilent dual-color microarrays (data obtained from the MAQC dataset [6]). We selected this experiment specifically, because treatment and array effects are not partially confounded (as is the case in the cell line dataset), and biological variance is absent. We observed a very strong linear correlation between real and *in silico *reconstructed ratios (Additional file 3), and the ANOVA-derived array effect was very small compared to the treatment effect (Figure 2B).

**Figure 3:**
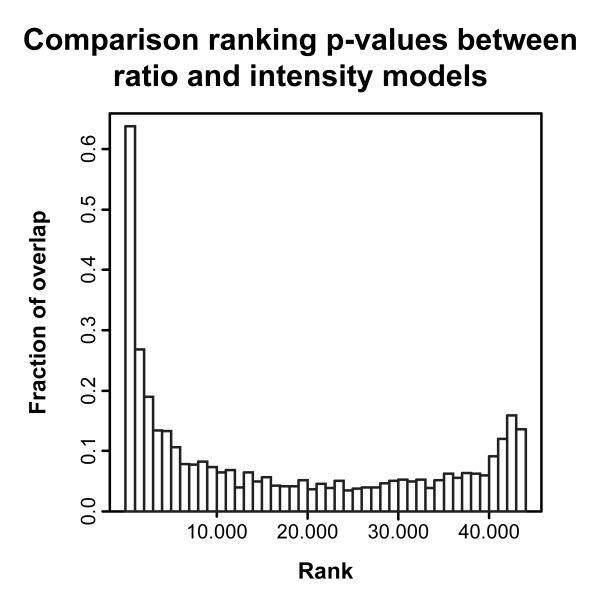
**Ratio- and intensity-based analysis results in similar sets of differentially expressed genes**. For the cell line dataset C1, p-values generated by the ratio and intensity ANOVA models were ranked from low to high, and assigned to bins containing 1000 genes. The fraction of overlap represents the proportion of genes occurring in both sets.

Ultimately, the reliable detection of differential gene expression between treatments is the main interest of the cell line experiment. We therefore, for the cell line dataset, compared the ranking of genes by p-values generated with two different ANOVA models: one including the array effect (the ratio analysis), and one without the array effect (the intensity analysis). We observed a substantial overlap of 64% (replicate dataset C1) and 66% (replicate dataset C2) between the ratio- and intensity-based lists of the 1,000 most significant genes, suggesting that the intensity-based model yields similar results as the ratio-based model (Figure 3). Furthermore, when using the p-value of the 1,000th most significant gene in the ratio dataset as a cutoff for the intensity dataset, 89% (dataset C1) and 92% (dataset C2) of the 1,000 genes selected by the ratio model were also present in the set of intensity-selected genes. Interestingly, the p-values generated by the intensity model are smaller than those generated by the ratio model, indicating that the intensity model is more sensitive in detecting differential gene expression.

### Intensity-based results reproduce better than ratio-based results

**Figure 4:**
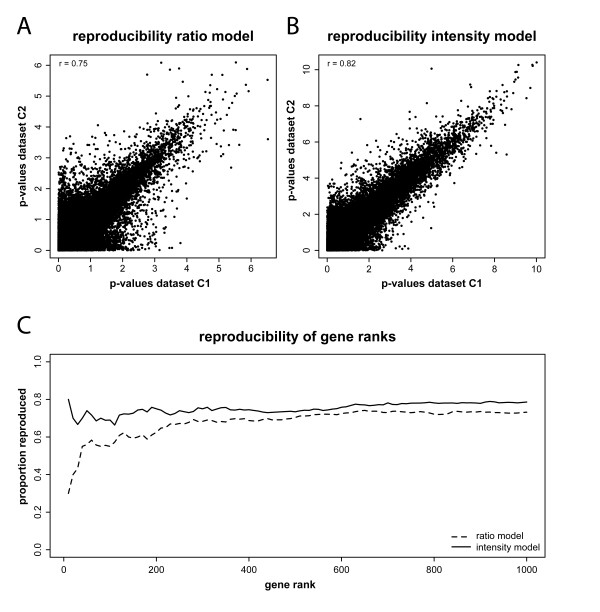
**Intensity models provide more reproducible results than ratio models**. A, B) Comparison between the reproducibility of p-values between technically duplicated experiments, generated by the ratio model (A) and the intensity model (B). Note the higher correlation for the intensity model. p-values are given as -log10(p-value): higher p-values are more significant. C) proportion of genes reproduced by either the ratio on intensity model, for sets of equally ranked genes between the replicate datasets C1 and C2.

The detection of regulated genes should be reproducible; between two technically replicated experiments, one expects to find highly similar sets of differentially expressed genes. To assess the reproducibility of the ratio and intensity models, we separately calculated p-values for both technical replicate datasets C1 and C2 of the cell line experiment. We observed a strong correlation (r = 0.75) between p-values generated by the ratio model for the two datasets (Figure 4A). However, the correlation between the p-values generated by the intensity model was even more pronounced (r = 0.82, Figure 4B). Next, we compared, for increasing numbers of genes, the overlap between the highest ranked genes within the replicate datasets, based on p-values generated either by the ratio- or intensity based ANOVA models (Figure 4C). Regardless of the size of the top-ranked gene lists (n = 10-1,000, increments of 10), the intensity model outperforms the ratio model: more genes are reproduced by the intensity model.

The results from the p-value rank-based reproducibility experiments suggest that it is preferable to exclude the array factor in the linear model. To provide further evidence that the intensity model is indeed superior to the ratio model, we applied the Bayesian information criterion (BIC) for model selection on the ratio- and intensity-based ANOVA models [8]. This test does not directly compare the outcomes of the intensity- and ratio-based analysis, but rather determines which of the two analyses is most suitable to analyze the data. A BIC calculation between the ratio and intensity models was performed for each gene. Indeed, for 94.5% of the genes, the intensity model is favorable over the ratio model, as determined by lower BIC values. For 5.5% of the genes, the inclusion of an array effect in the linear model resulted in lower BIC values (Table 1).

**Table 1:** The intensity model is favored over the ratio model based on BIC model selection.

	Ratio	Intensity	Total
***Cell line dataset***			
Genes	2382	40976	43358
Percentage	5.49%	94.51%	100.00%
			
***Brain dataset***			
Genes	4	39413	39417
Percentage	0.01%	99.99%	100.00%

Results of the per-gene BIC model selection for both the cell line and human brain datasets. The column *Ratio *represents the number or percentage of genes with lower BIC values as opposed to the intensity model. The *Intensity *column represents the number or percentage of genes with lower BIC values as opposed to the ratio model.

### Analysis of an independent dataset confirms the gain in reproducibility and sensitivity when using intensity-based models

**Figure 5:**
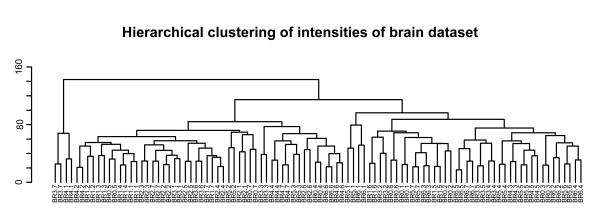
**Single channel clustering of human brain dataset**. Hierarchical clustering of log2-transformed single channel intensities of the human brain experiment. Only genes with an average intensity A > 7 were used. Note that, for all 49 individuals, the two replicate measurements cluster together.

The cell line experiments demonstrated that intensity-based analysis of dual-color data provide more reproducible results, and is more sensitive in the detection of differentially expressed genes. It is however unknown how these results translate to other types of experiments, consisting of different sample types and experimental setups. We therefore analyzed a separate dataset, consisting of 49 human prefrontal cortex samples, divided over 7 equally sized groups. Samples were obtained from different human subjects, thus the biological variance in this dataset is expected to be large (no biological variation is present in the cell line dataset). Consequently, samples were not pooled, but hybridized individually. As each biological sample was hybridized two times (Additional file 4), we expected the distribution of intensities from the two separate hybridizations to be very similar. Indeed, as previously observed in the cell line experiments, an unsupervised hierarchical clustering of all single channels showed that the two intensity measurements of the same biological sample clustered together (Figure 5).

**Figure 6:**
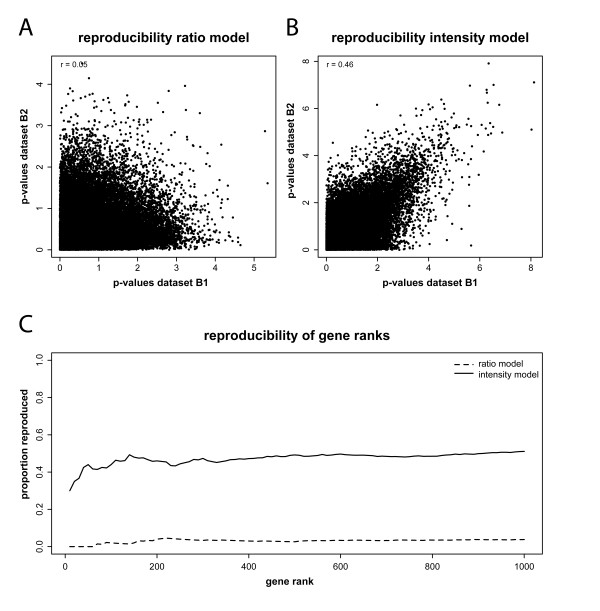
**Comparison between ratio and intensity model-based reproducibility in the brain dataset**. A, B) Comparison between the reproducibility of p-values between the split brain datasets B1 and B2, generated by the ratio model (A) and the intensity model (B). Note the absence of correlation between p-values calculated with the ratio model. p-values are given as -log10(p-value): higher p-values are more significant. C) proportion of genes reproduced in sets of equally ranked genes between the replicate datasets.

The human brain experiment was not designed with full technical replication in mind. However, as we performed duplicate measurements for each sample, it was possible to divide the dataset into 2 identical biological datasets B1 and B2 (see Additional file 4). To assess the reproducibility between these two replicate datasets, we again compared the p-values generated both by the ratio and the intensity models. We found a surprising lack of correlation (r = 0.05) for p-values based on the ratio model between replicates (Figure 6A). The p-values generated by the intensity model however showed a far better correlation (r = 0.46, Figure 6B). Indeed, when determining, for increasing numbers of genes (10-1,000 genes, increments of 10), the overlap between the highest ranked genes based on the p-values of the replicate measurements of either the ratio- or intensity based ANOVA models, we observed a substantial proportion of reproduced genes in the intensity model. Almost no genes were reproduced between the two ratio-based analyses (Figure 6C). Not surprisingly, model selection according to the BIC showed that for 99.99% of the genes, the intensity model outperforms the ratio model. For only 4 genes, the array component was large enough to justify incorporation in the ANOVA model (Table 1). Thus, also in this second experimental dataset, there is clear evidence that the intensity model is to be preferred over the ratio model.

**Figure 7:**
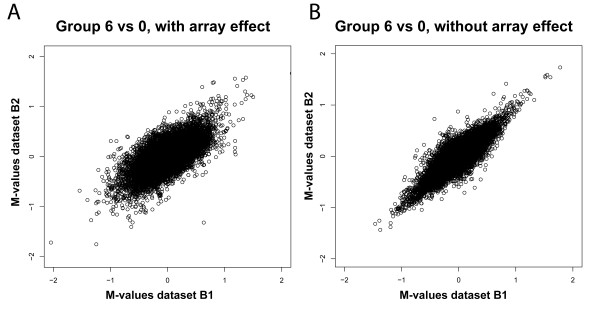
**Reproducibility of between-group treatment effects based on ratio and intensity models**. Reproducibility of ANOVA-derived treatment effects between group 0 and group 6 in replicate brain datasets B1 and B2. Panel A: reproducibility of treatment effects derived from the ratio model. Panel B: reproducibility of treatment effects derived from the intensity model. Note the enhanced reproducibility when using the intensity-based ANOVA model.

As the brain dataset is based on human subjects, the biological variation is large, which is reflected in overall less significant p-values than the cell line dataset. Consequently, replication on the p-value level is less pronounced than for the cell line dataset. We therefore also analyzed replication in the human brain dataset on the treatment effect level. For the two replicate datasets, the size of the treatment effect between different pairwise sample group comparisons (positioned at different distances in the loop design) was estimated using ANOVA models with and without the array factor (Figure 7 and Additional file 5). In all comparisons, the intensity-based analysis resulted in better correlations between M-values from the two replicate datasets.

### A power perspective on intensity versus ratio-based models

**Figure 8:**
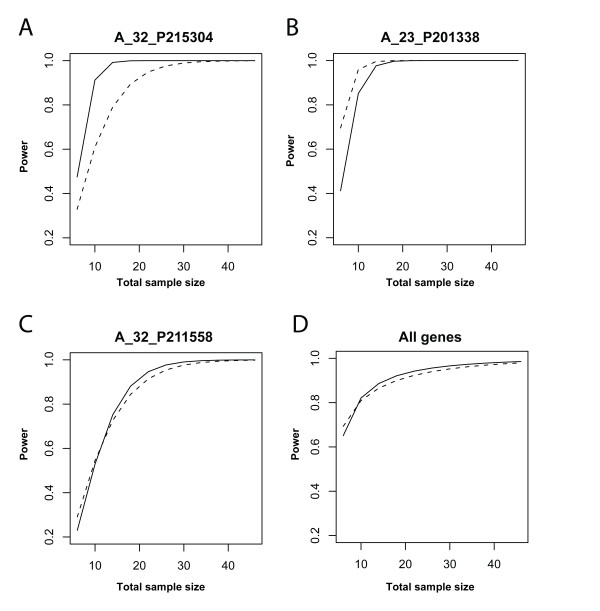
**Power curves for intensity and ratio models for the MAQC dataset**. A-C) Power curves for probes A_32_P215304, A_23_P201338 and A_32_P211558 using treatment effect sizes equal to their estimated values, -0.34, -0.35, -0.50. D) Average power for all probes in the MAQC dataset. Only probes with an estimated effect size larger than 0.25 were taken into account. Solid line: model including array effect; dashed line: model without array effect.

In both the cell line and human brain datasets, the factors treatment and array are partially confounded (since one cannot assign every treatment to each array). In the MAQC dataset, which consists of 10 technical replications of two commercially available RNA samples, these factors are not confounded. A BIC analysis revealed that the model without array effect is preferable for 71% of the genes in the MAQC dataset. Thus, even though dropping the array effect is beneficial for roughly 2 out of every 3 genes, this percentage is lower than in the other two data sets. This is however not unexpected: BIC includes a penalty which is proportional to the sample size (number of arrays) for the model with array effect while constant for the other model, and the sample size of the MAQC dataset is smaller than those of the other two data sets. The simple repeated measurements design of the MAQC data set allowed us to study the trade-off between less degrees of freedom and variance reduction caused by inclusion of the array effect from a power perspective. Figure 8A-C shows the power curves for probes A_32_P215304, A_23_P201338 and A_32_P211558, assuming that the estimated treatment effect sizes are real (the treatment effect sizes are the same for both the ratio and intensity models). While one analysis may dominate the other for all sample sizes (Figure 8A and 8B), we observe the aforementioned trade-off from the crossing curves in Figure 8C. In all cases the power curve for the analysis including the array effect is steeper, confirming our expectations that when sample size increases, the loss of degrees of freedom is less harmful. We also considered the average power (the expected number of genes declared significant) over the entire MAQC dataset (Figure 8D). The gene set was restricted to those with an estimated treatment effect size larger than 0.25 to emulate a set that contains differential signal. The average power was higher for the model with array effect for total sample size larger or equal to 12 while smaller otherwise. Differences were small, though: a maximum difference of 1% was found. As expected the power curves converge again when the sample size increases. As opposed to the reproducibility results, these power calculations assumed the array effects to be fixed, as implied by the model. While this may be too optimistic, it is good to notice that the aforementioned trade-off is visible from a power perspective.

## Discussion

Our results demonstrate that the analysis of dual-color microarray gene expression experiments using intensity-based linear models outperforms the standard ratio-based analysis. Both reproducibility and sensitivity were enhanced in detecting differential gene expression in two independent datasets.

By analyzing technically replicated experiments we determined the effect of both models on the reproducibility of gene rankings. Our studies show that for both the cell line and brain datasets the intensity-based analysis provides more reproducible gene rankings than the ratio-based analysis of the same dataset. For the cell line dataset, 78% of the 1,000 most significant genes is reproduced between the two duplicate datasets C1 and C2 when using the intensity analysis, whereas only 73% of the genes is reproduced with the ratio analysis (see Figure 4C). For the brain datasets B1 and B2, the difference between ratio- and intensity-based reproducibility is far more pronounced: only 4% of the top 1,000 genes are reproduced in the ratio analysis, while there is still a substantial overlap of 51% between intensity-based gene rankings (Figure 6C). The underlying reasons behind the apparent discrepancy between the cell line and brain datasets will be addressed later. An independent line of evidence, based on model selection, also indicated that intensity-based models are preferred over ratio-based models for the analysis of dual-color microarray data. When performing Bayesian Information Criterion model selection calculations, we found that for 95% of the transcripts in the cell line experiment, and virtually all transcripts in the human brain experiment, the intensity model was favored over the ratio model. Furthermore, for both the cell line dataset and a publicly available third dataset, a comparison between ANOVA-based array and treatment effect sizes revealed that the treatment effects are much larger.

Combining the gene ranking, relative effect size and model selection results, we argue that simply by selecting the intensity model instead of the ratio model for the analysis of the same set of gene expression measurements, more reproducible results are obtained.

It should be noted that the relative advantage of dropping the array effect depends on the complexity of the design and the sample size (the number of arrays). For the relatively simple MAQC data set BIC selects the model with array effect for 29% of the genes, much more frequently than for both the brain and cell line data sets. The beneficial effect of dropping the array effect from the model seems more pronounced in experiments that employ direct designs to address complex comparisons, such as time series and multifactorial experiments.

Adding to the enhanced reproducibility, intensity-based analysis is more sensitive in the detection of differential gene expression, as derived from more significant p-values. It is important to note that, by selecting the ratio-based p-value of the 1000^th ^most significant gene as a cutoff, almost all of the 1000 genes (89% for dataset C1, 92% for dataset C2) are also significant in the intensity-based analysis using the same cutoff. Interestingly, this analysis also reveals that 3335 genes, not selected by the ratio model, are reproducibly more significant than the 1000^th ^gene in the ratio results. This provides additional evidence for the enhanced sensitivity of the intensity model over the ratio model. Due to the poor reproducibility of the ratio-based results in the brain dataset, such calculations were not meaningful for that dataset.

### Enhanced sensitivity due to ignoring the array effect in the linear model

The observation that ratio-derived p-values can be improved by intensity-based models can be attributed to the inclusion of the array effect in the ratio-based linear model. Pairing of data is a powerful concept for removing subject specific bias. In particular, when the quality of the spot printing procedure is not constant (often the case with in-house spotted arrays), it is essential to account for an array effect in the ANOVA model [9]. But there is a price to pay: degrees of freedom [10]. The total number of degrees of freedom equals the number of samples. The array effect consumes almost half of the degrees of freedom. However, due to the high quality of commercially available dual-color oligonucleotide microarrays, we and others observed that the ratios of the same sample pair, measured on different arrays, are strongly correlated [6], which means that the array effect is likely to be very small. When using a ratio-based model to analyze the data, many degrees of freedom are used to estimate the array effect, explaining only a small proportion of the variability. This ultimately results in less significant p-values, a lower correlation between p-values from the two replicate experiments, and a smaller proportion of reproduced top-ranked genes. Indeed, the results from the model selection experiments clearly indicate that the model without array effect is the preferred model for both datasets. It should be noted that we do not state that the array effect is absent: our analyses in fact show that an array effect is present in modern dual color microarray experiment. Furthermore, the results from the power calculations for the MAQC dataset show that including the array effect can be slightly beneficial for certain sample sizes. However, we conclude from our experiments that for both the brain and cell line datasets, the array effect is too small in comparison to the main factor of interest (treatment) to justify incorporation into the ANOVA model.

A possible argument for the inclusion of the array effect is the potential competition for spot binding between the co-hybridized samples. However, our and other studies suggest that competition is not an issue [7,10]. This can be derived from the strong correlation between the real and *in silico *reconstructed ratios (see Additional files 2 and 3), and the hierarchical clustering in Figures 1 and 5. Our study was however not conducted to demonstrate that ratios can be reconstructed *in silico *by using separate intensities. Indeed, this has been demonstrated before [10]. Our specific aim was to compare the performance of ratio- and intensity-based methods based on the main outcome of comparative gene expression experiments: a list of ranked genes. As this gene ranking provides the basis for further research, it needs to be robust and reproducible. We show here that intensity-based methods provide more reproducible results and is more sensitive in detecting differential gene expression, and thus outperform the standard ratio-based analysis.

### Biological variation negatively affects ratio-based, but not intensity-based, replication

As indicated earlier, in the human brain experiment, we observed a striking lack of reproducibility (r = 0.05) between p-values generated by the ratio model on the replicate datasets B1 and B2, whereas the intensity-based p-values reproduced quite well (r = 0.46). These findings can be attributed to the following. First of all, the overall p-values (both intensity- and ratio-based) are less significant in the human brain experiment than in the cell line experiment, due to the large biological variation between individuals. Second, due to the relatively low level of biological replication, few degrees of freedom are left for estimating the biological effect. Third, the brain experiment was not designed with splitting the data into two technical replicates in mind. While the two data sets are biologically identical, the samples are paired differently on the arrays between the two replicate datasets (see Additional file 4). Since this pairing is more or less arbitrary, the results should be robust against this artifact, but this is not necessarily the case for the ratio-based analysis. When the biological variation is large, different sample pairings may result in differences in measured ratios, a phenomenon we observed in the brain dataset (Figure 7 and Additional file 5). The intensity-based analysis of brain datasets B1 and B2 does not suffer from these drawbacks: no ratios are calculated, and more degrees of freedom are left for estimating the biological effect of interest, resulting in a substantial proportion of reproducible findings (51% of the 1,000 most significant genes), and a relatively high correlation between p-values (r = 0.46). In a setting with many biological replicates per level (e.g. comparison of two large groups) the differences in correlation between the ratio-based and intensity-based analysis are likely to be smaller.

Our studies indicate that the reliability of gene rankings obtained from dual-color microarray experiments can be improved by using intensity-based models. An added benefit of the intensity-based analysis is that intensity models do not suffer from the drawbacks of ratio models in the analysis of complex direct dual-color experiments. Designs such as the interwoven loop design address the increased complexity of microarray experiments, which have progressed from "simple" two-group comparisons to multifactorial or time-course experiments. The aforementioned direct designs are efficient, but often bias certain comparisons over others and lack the possibility to extend the experiment by adding more groups or samples. There are no such limitations when analyzing dual-color experiments with intensity-based models [10]. Finally, the LIMMA software package also uses intensity data from dual-color experiments, but mainly as a solution to compare samples which are unconnected in the hybridization design [11]. Here, we provide evidence that it is beneficial to perform an intensity-based analysis for connected designs as well. It should be noted that the observed improvements may be limited to dual-color arrays and that further experiments are needed to justify the generality of these results for other array designs.

## Conclusions

In conclusion, our results indicate that intensity-based models are very powerful in the analysis of dual-color gene expression data when these are obtained from a high-quality platform. Most importantly, intensity models yield more reproducible results, and are more sensitive in the detection of differential gene expression than standard ratio-based analysis methods on the same microarray dataset. The gain in reproducibility and sensitivity are most pronounced in complex designs such as the interwoven loop design. We argue that the intensity-based models outperform ratio-based models, and thus are the preferred models for the analysis of dual-color gene expression datasets derived from commercial oligo-based array platforms.

## Methods

### Human keratinocyte cell line dataset

The cell line sample set consisted of two immortalized cell lines (cell lines 10 and 19) derived from a single primary keratinocyte culture. The two cell lines were subjected to four different treatments (treatments T1, T2, T3 and T4). After RNA isolation and labeling (labeling performed with Agilent Low RNA Input Fluorescent Linear Amplification Kit, Agilent Technologies), equal amounts (1 μg) of Cy3-CTP and Cy5-CTP labeled samples were hybridized to Agilent 4 × 44K Whole Human Genome arrays (Agilent Technologies, Part Number G4112F), according to the manufacturer's instructions. The hybridization set-up on the 4 × 44K array was chosen in such a way that for each cell line, both Cy3- and Cy5-labeled samples for all treatments were hybridized on a single slide (containing 4 arrays). The entire experiment was technically replicated. The hybridization setup can be found in Additional file 1. Microarrays were scanned using the Agilent DNA Microarray Scanner (Agilent Technologies, Part Number G2505B), and scans were quantified using the Agilent Feature Extraction software (version 8.5.1). Raw expression data generated by the Feature Extraction software were imported into the R statistical environment using the LIMMA package [12] in Bioconductor http://www.bioconductor.org. No background correction was performed, as overall background levels were very low. The intensity distributions within and between arrays were normalized using the *quantile *scaling algorithm [13] in LIMMA. After normalization, the separate intensity channels were extracted from the ratio measurements. The log2-transformed intensity measurements were used in all following analyses. The microarray data have been deposited in the Gene Express Omnibus (GEO) database http://www.ncbi.nlm.nih.gov/geo/query/acc.cgi?token=djkxjoomeyecqja&acc=GSE12553.

### MAQC dataset

Microarray hybridization data were extracted from the Gene Expression Omnibus (GEO accession number GSE5350, file MAQC_AGL_123_60TXTs.zip, series "C": arrays AGL_3_C1.txt, AGL_3_C2.txt, AGL_3_C3.txt, AGL_3_C4.txt and AGL_3_C5.txt, series "D": AGL_3_D1.txt, AGL_3_D2.txt, AGL_3_D3.txt, AGL_3_D4.txt and AGL_3_D5.txt). This dataset consists of 10 technical replications of a hybridization of Stratagene Universal Human Reference RNA (Cy3 in series "C", Cy5 in series "D") and Ambion Human Brain Reference RNA (Cy3 in series "D", Cy5 in series "C") as described in [6]. Microarray normalization procedures were performed as described for the cell line experiment. Power curves were computed from the non-central t-distribution.

### Human brain dataset

Fresh-frozen human brain tissue samples were obtained from the Netherlands Brain Bank, Amsterdam (NBB). Written informed consent for a brain autopsy and the use of the material and clinical information for research purposes was obtained by the NBB from the donor or next of kin. Gray matter was isolated from the prefrontal cortex of 49 individuals (matched for age, sex, postmortem interval and brain pH) with increasing levels of AD-related neuropathology, as defined by the Braak staging for neurofibrillary tangles [14]. For each of the 7 Braak stages, 7 individuals were included. Tissue dissection was performed using a cryostat. For each sample, between 20 and 30 sections of 50 μm were cut. Grey matter areas were identified by eye and dissected out using pre-chilled scalpels. Tissue yields were typically around 50 mg. Total RNA was isolated using a combination of Trizol-based and RNeasy Mini Kit RNA isolation methods. Briefly, samples were homogenized in ice-cold Trizol (Life Technologies, Grand Island, New York, 3 ml Trizol per 100 mg tissue). After phase separation by addition of chloroform, the aqueous phase was mixed with an equal volume of 70% RNAse-free ethanol. Samples were then applied to an RNeasy Mini column (Qiagen, Valencia, California), and processed according to the RNeasy Mini Protocol for RNA Cleanup. Overall, the isolated RNA was of high integrity (average RNA integrity number of of 8.3, range 6.5-9.6, as determined by Agilent 2100 Bioanalyzer analysis).

After RNA isolation, for each sample, two 500 ng aliquots of RNA were linearly amplified and fluorescently labeled with either Cy3-CTP or Cy5-CTP (Perkin Elmer) with the Agilent Low RNA Input Fluorescent Linear Amplification Kit (Agilent Technologies). The most efficient hybridization scheme was calculated with the *od *function of the SMIDA package (version 0.1) in R. The resulting hybridization setup can be found in Additional files 4 and 6. Equal amounts (1 μg) of Cy3-CTP and Cy5-CTP labeled samples were hybridized to Agilent 44K Whole Human Genome arrays (Part Number G4112A) according to manufacturer's instructions. Microarray scanning, feature extraction and normalization procedures were performed as described for the cell line experiment. The full set of normalized expression values is publicly available at http://www.vumc.nl/braindataset and as supplementary information to this manuscript (see Additional file 7).

### Clustering and ANOVA models

Clustering of the intensity channels was performed using complete linkage hierarchical clustering. Only probes for which the average log2-transformed intensity (A, as derived from the separate Cy3 or Cy5 channels) was above A = 7 were included. As this procedure removes data from all arrays for a particular probe, the sample sizes are the same for each probe in the final dataset. For the cell-line data, p-values for differential expression between treatments were generated as follows. First, the entire data set was split into two biologically identical parts by simply distinguishing the technical replicates A and B (Additional file 1). The split resulted in cell line data sets C1 and C2. Next, two types of ANOVA models were used per array element. Model 1 represents the ratio-based analysis:(1)

Here, *μ *captures the average gene intensity, τ_*i *_is the treatment specific effect, *η*_*j *_is the cell line (10 or 19) effect, *α*_*k*(*j*) _is the array effect, and *ε*_*ijk *_is the error component. Dye effects have not been incorporated, because the design was balanced for dyes and the data were normalized to remove dye-specific bias. Model 2 lacks the factor *α*_*k*(*j*) _and hence represents the intensity-based model. The treatment effect is the factor of biological interest, to which an F-test was applied to compute p-values. This resulted in four lists of p-values: ratio-based and intensity-based p-values for technical replicate C1, and ratio-based and intensity-based p-values for technical replicate C2.

A similar approach was taken for the human brain data. Each patient was hybridized twice. The resulting set of arrays was split in such a manner that each patient was represented exactly once in both data sets (brain datasets B1 and B2, see Additional file 4). It is noteworthy that the obtained datasets are indeed technical replicates, but not on the level of the experimental design, as is the case for cell line datasets C1 and C2. The cell line effect *η*_*j *_was dropped from the model, and the treatment effect τ_*i *_now represented the Braak stage. The F-test was performed on the Braak stage factor. Again, two ANOVA models were used: the ratio model which included the array effect *α*_*k*(*j*)_, and the intensity model without array effect. Consequently, four lists of p-values were generated: ratio-based and intensity-based p-values for dataset B1, and ratio-based and intensity-based p-values for dataset B2.

We did not apply any multiple testing corrections for our purpose, since a criterion like False Discovery Rate (FDR) might distort the comparison between the models somewhat. Also, since both splits contain an equal numbers of samples, sample size 'bias' is absent.

### Comparison ratio and intensity data, reproducibility calculations and model selection

For the cell line dataset, direct ratio measurements between co-hybridized sample pairs were compared with *in silico *reconstructed ratios of the two intensity measurements of the same sample pair, as measured on separate arrays, and against different samples. For example, in dataset C1, the directly measured ratios between samples T1 and T2 on array 1, were compared with the reconstructed ratios between T1 on array 4, and T2 on array 2 (Additional file 1). To eliminate possible confounding effects of noise introduced by genes expressed at very low levels, only genes with an average log-transformed intensity levels greater than 7 were used. To compare the overlap between gene rankings based on the ratio and intensity models, genes were ordered by p-value and assigned to bins containing 1,000 genes. The fraction of overlap then was defined as the amount of genes ranked in the same bin by both models, divided by the size of the bin.

Both for the cell line datasets C1 and C2, and the human brain datasets B1 and B2, reproducibility between the replicated datasets was determined as follows. First, the correlation between sets of p-values was calculated using Spearman's *rho*. Second, to assess the proportion of genes with similar ranks between replicates, genes were ordered by p-value. For bins of increasing size (10 to 1,000 genes, by increments of 10 genes), the proportion of overlap was defined as the fraction of genes, occurring in both sets. Third, Bayesian information criterion (BIC) model selection was used to score the ratio- and intensity-based linear models for each array feature. Information criterion methods, which aim to determine which set of model parameters the data support best, penalize models with more unknown parameters in order to select a model with a lower generalization error and hence more reproducible results [15]. The preferred model was defined by the model with the lowest BIC value. BIC calculations were performed using the *nlme *package in R.

## Competing interests

The authors declare that they have no competing interests.

## Authors' contributions

KB generated the human brain dataset, conceived of the study, performed statistical analyses, and drafted the manuscript. RHB and SJS generated the human keratinocyte cell line dataset. BY and JV participated in the design of the study and helped to draft the manuscript. MAW conceived of the study, performed statistical analyses and helped to draft the manuscript. All authors read and approved the final manuscript.

## Supplementary Material

Additional file 1 **Hybdridization setup cell line experiment**. DN = P53DN mutant, SH= shRNA, MI = MIR372, E6 = HPV16 E6. The array numbers are given in the top left corner of each array. Click here for file

Additional file 2 **Correlation real and in silico reconstructed ratios**. Correlation between ratios, as measured on the array, and the in silico reconstructed ratios of the same sample pair, as measured on different arrays and against different co-hybridized samples. Only genes with an average log2-transformed expression > 7 were included. Click here for file

Additional file 3 **Correlation real versus virtual hybridizations**. Correlation between M-values derived from real hybridizations versus M-values derived from virtual hybridizations for the MAQC dataset. Main title: hybridization number. X-axis: directly measured ratio between sample A and B. Y-axis: mean M-value between 9 virtual comparisons sample A versus sample B, where sample A and B where measured on different arrays. Correlation: Pearson's correlation coefficient. Click here for file

Additional file 4 **Hybridization setup human brain experiment**. The column *dataset *denotes in which replicate dataset (B1 or B2) the intensity measurement was used. Click here for file

Additional file 5 **Reproducibility of between-group treatment effects based on ratio and intensity models**. Reproducibility of ANOVA-derived treatment effects between group 2 and group 4, and group 1 and group 5, in replicate brain datasets B1 and B2. Reproducibility is enhanced when using intensity-based models instead of ratio-based models. Click here for file

Additional file 6 **Hybdridization setup brain experiment**. BR = Braak stage. Click here for file

Additional file 7 **Human keratinocyte cell line dataset**. Normalized expression values and raw hybridization data for all hybridizations have been deposited into the Gene Expression Omnibus (GEO) database. Reviewer link GEO dataset: http://www.ncbi.nlm.nih.gov/geo/query/acc.cgi?token=djkxjoomeyecqja&acc=GSE12553. Click here for file

## References

[B1] Blalock EM Geddes JW Chen KC Porter NM Markesbery WR Landfield PW Incipient Alzheimer's disease: microarray correlation analyses reveal major transcriptional and tumor suppressor responses Proc Natl Acad Sci USA 2004 101 2173 2178 10.1073/pnas.0308512100 14769913 PMC357071

[B2] Bossers K Meerhoff G Balesar R van Dongen JW Kruse CG Swaab DF Verhaagen J Analysis of gene expression in Parkinson's disease: possible involvement of neurotrophic support and axon guidance in dopaminergic cell death Brain Pathol 2009 19 91 107 10.1111/j.1750-3639.2008.00171.x 18462474 PMC8094761

[B3] White KP Rifkin SA Hurban P Hogness DS Microarray analysis of Drosophila development during metamorphosis Science 1999 286 2179 2184 10.1126/science.286.5447.2179 10591654

[B4] Blalock EM Chen KC Stromberg AJ Norris CM Kadish I Kraner SD Porter NM Landfield PW Harnessing the power of gene microarrays for the study of brain aging and Alzheimer's disease: statistical reliability and functional correlation Ageing Res Rev 2005 4 481 512 16257272 10.1016/j.arr.2005.06.006

[B5] Kerr MK Churchill GA Experimental design for gene expression microarrays Biostatistics 2001 2 183 201 10.1093/biostatistics/2.2.183 12933549

[B6] Patterson TA Lobenhofer EK Fulmer-Smentek SB Collins PJ Chu TM Bao W Fang H Kawasaki ES Hager J Tikhonova IR Performance comparison of one-color and two-color platforms within the MicroArray Quality Control (MAQC) project Nat Biotechnol 2006 24 1140 1150 10.1038/nbt1242 16964228

[B7] Buffart TE Israeli D Tijssen M Vosse SJ Mrsic A Meijer GA Ylstra B Across array comparative genomic hybridization: a strategy to reduce reference channel hybridizations Genes Chromosomes Cancer 2008 47 994 1004 10.1002/gcc.20605 18663753

[B8] Schwarz G Estimating the dimension of a model Annals of Statistics 1978 6 461 464 10.1214/aos/1176344136

[B9] Wolfinger RD Gibson G Wolfinger ED Bennett L Hamadeh H Bushel P Afshari C Paules RS Assessing gene significance from cDNA microarray expression data via mixed models J Comput Biol 2001 8 625 637 10.1089/106652701753307520 11747616

[B10] Hoen PA Turk R Boer JM Sterrenburg E De Menezes RX Van Ommen GJ Den Dunnen JT Intensity-based analysis of two-colour microarrays enables efficient and flexible hybridization designs Nucleic Acids Res 2004 32 E41 10.1093/nar/gnh038 14982960 PMC390313

[B11] Smyth GK Gentleman R, Carey V, Dudoit S, Irizarry RA, Huber W Limma: linear models for microarray data Bioinformatics and Computational Biology Solutions using R and Bioconductor 2005 397 420 full_text

[B12] Smyth GK Speed T Normalization of cDNA microarray data Methods 2003 31 265 273 10.1016/S1046-2023(03)00155-5 14597310

[B13] Bolstad BM Irizarry RA Astrand M Speed TP A comparison of normalization methods for high density oligonucleotide array data based on variance and bias Bioinformatics 2003 19 185 193 10.1093/bioinformatics/19.2.185 12538238

[B14] Braak H Braak E Neuropathological stageing of Alzheimer-related changes Acta Neuropathol (Berl) 1991 82 239 259 10.1007/BF00308809 1759558

[B15] Hastie T Tibshirani R Friedman J The Elements of Statistical Learning 2001 Springer-Verlag New York, LLC

