# Supplementary figures and images for: Intensity-based analysis of dual-color gene expression data as an alternative to ratio-based analysis to enhance reproducibility
Source: BMC Genomics. 2010 Feb 17;11:112. doi: 10.1186/1471-2164-11-112 (PMC2838842; doi:10.1186/1471-2164-11-112)

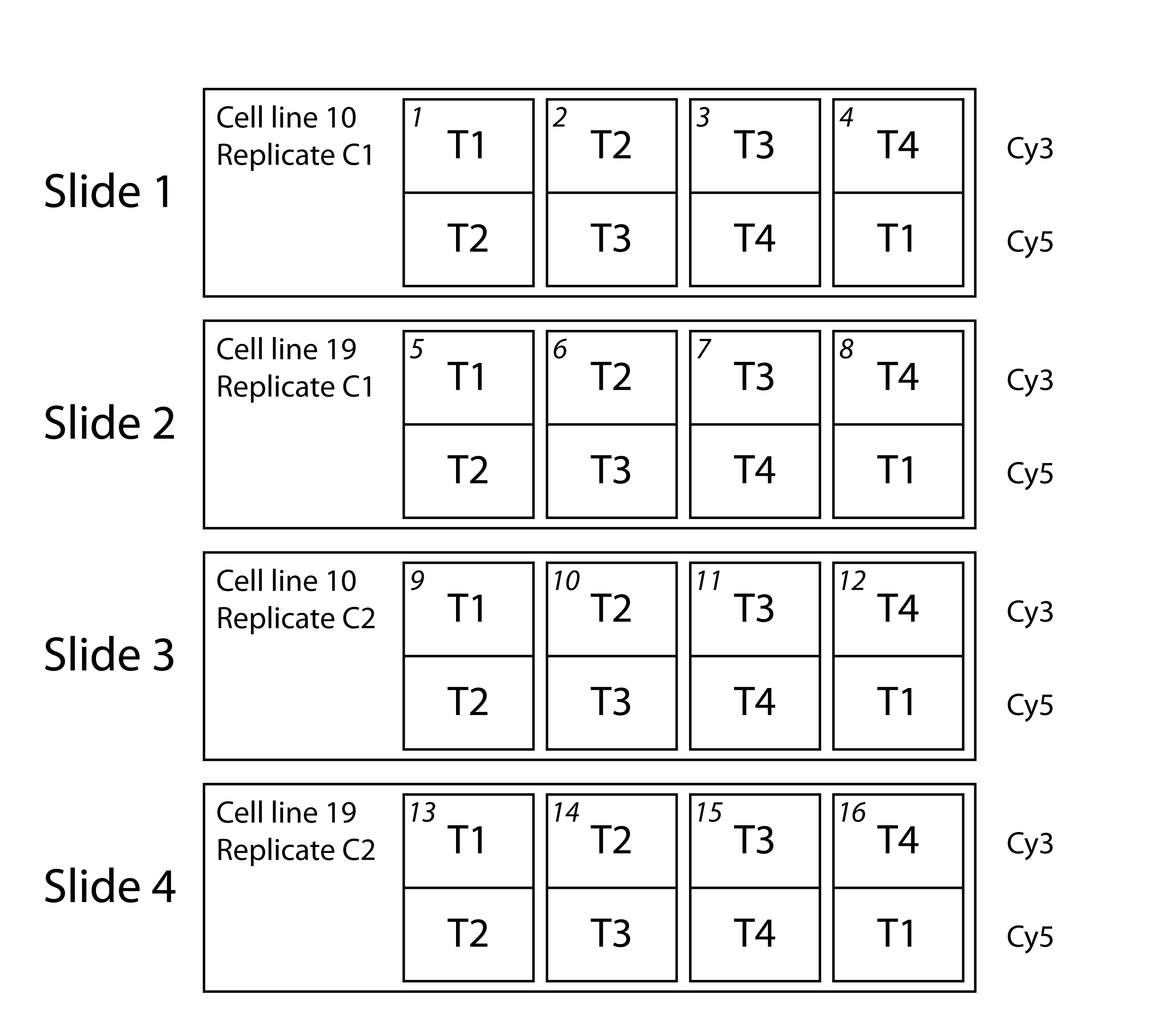

Supplement: Additional file 1 — Hybdridization setup cell line experiment. DN = P53DN mutant, SH= shRNA, MI = MIR372, E6 = HPV16 E6. The array numbers are given in the top left corner of each array. [file 1471-2164-11-112-S1.PNG]

C1

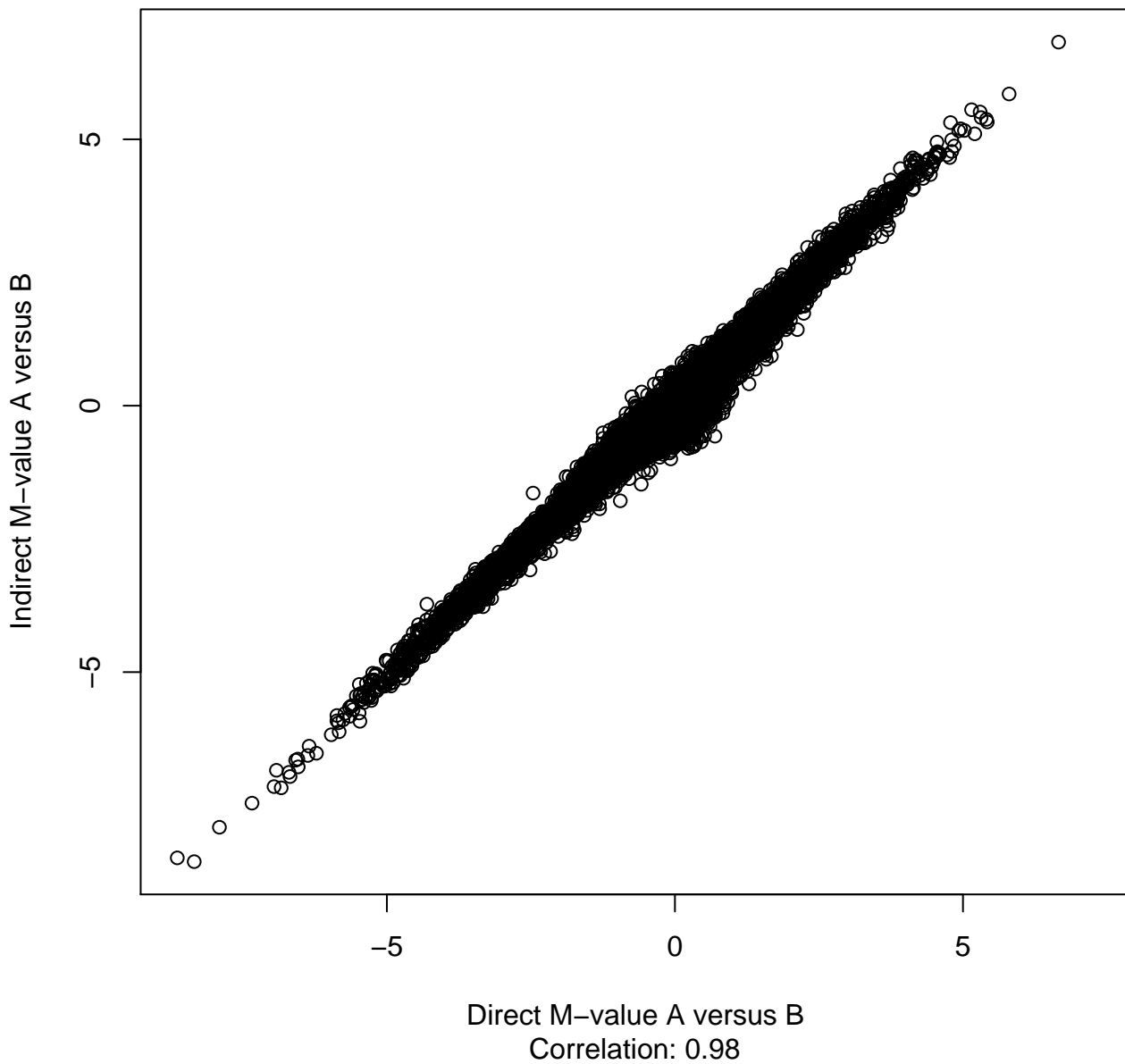

**C2**

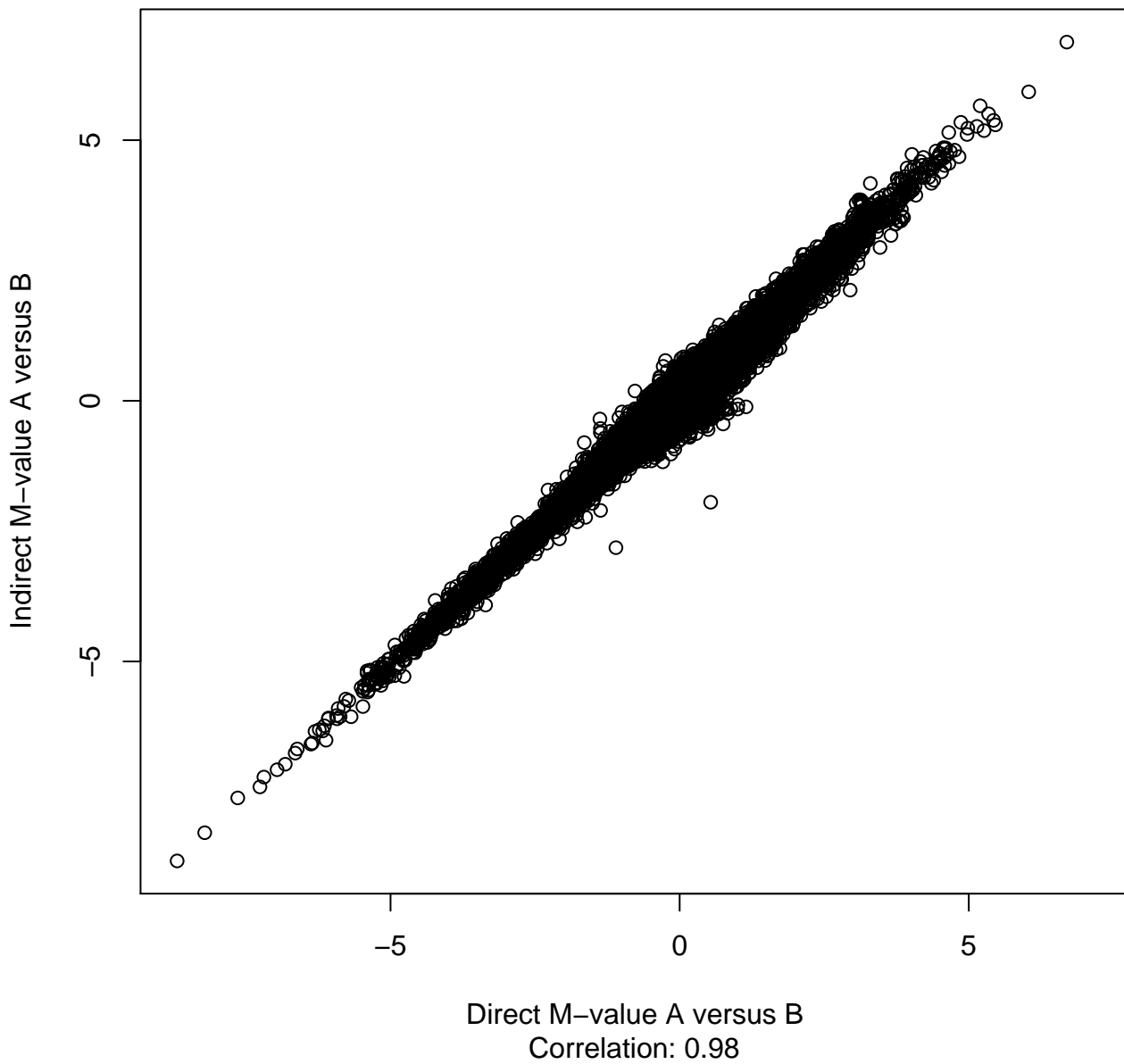

**C3**

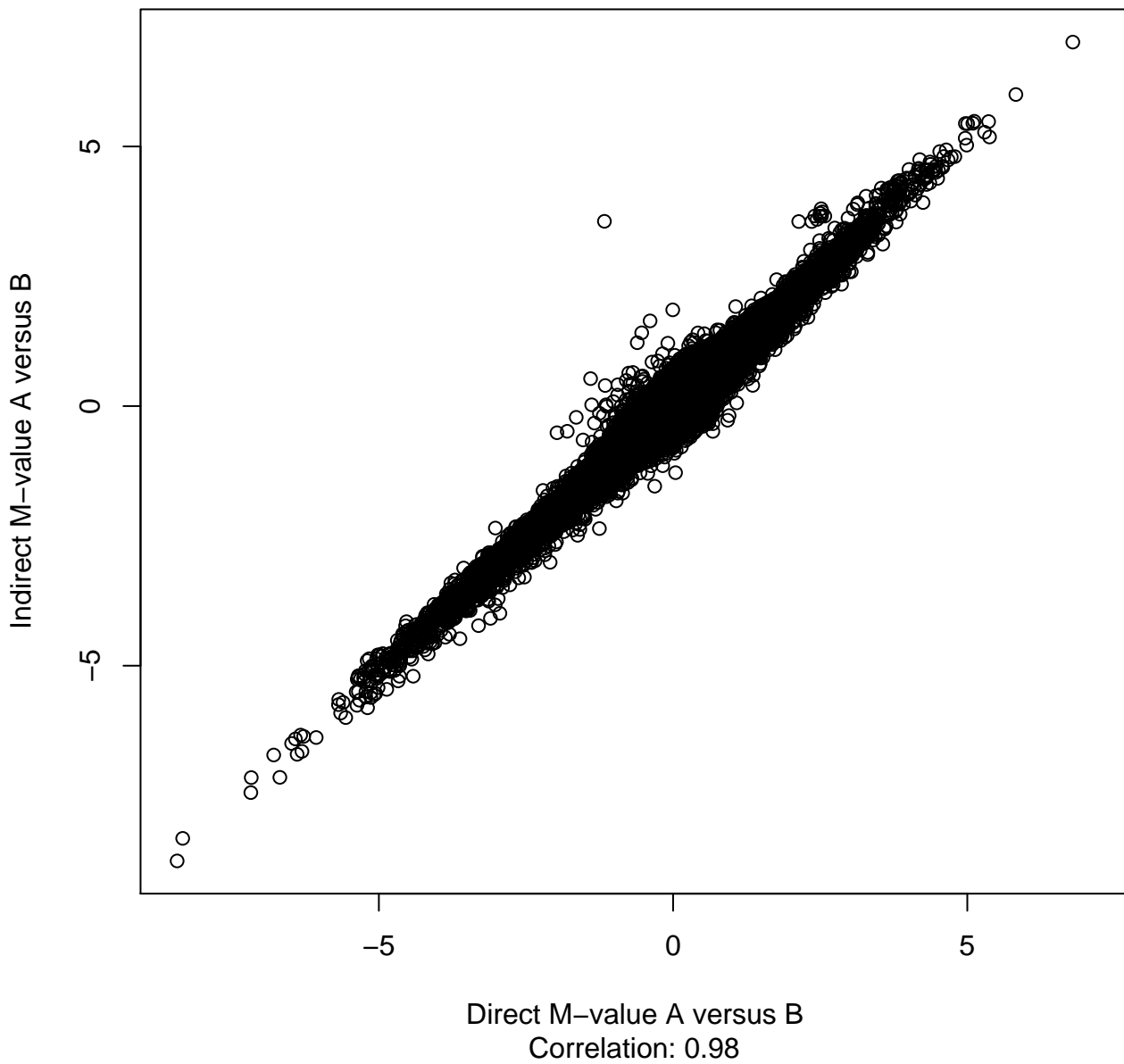

**C4**

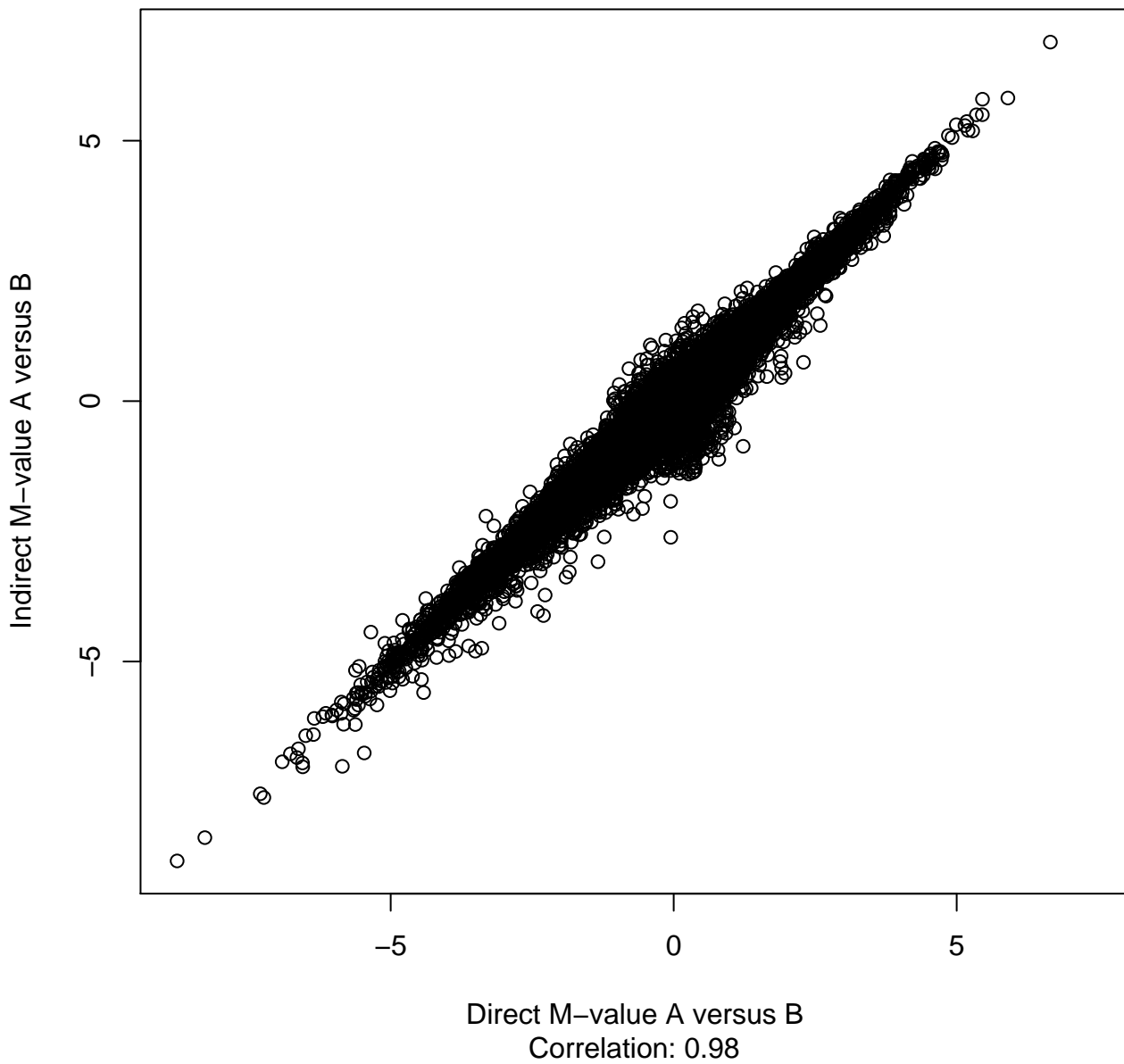

**C5**

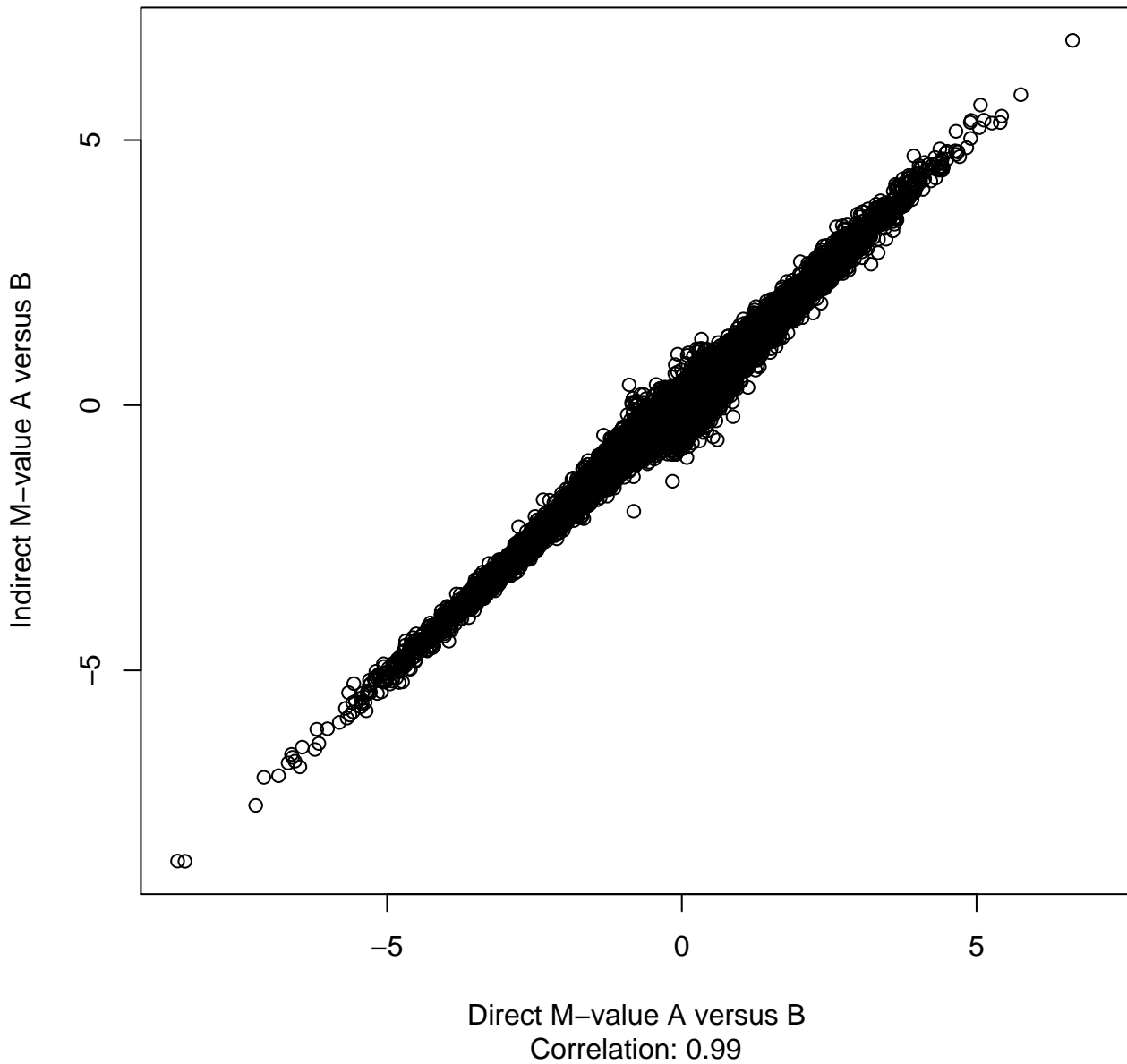

D1

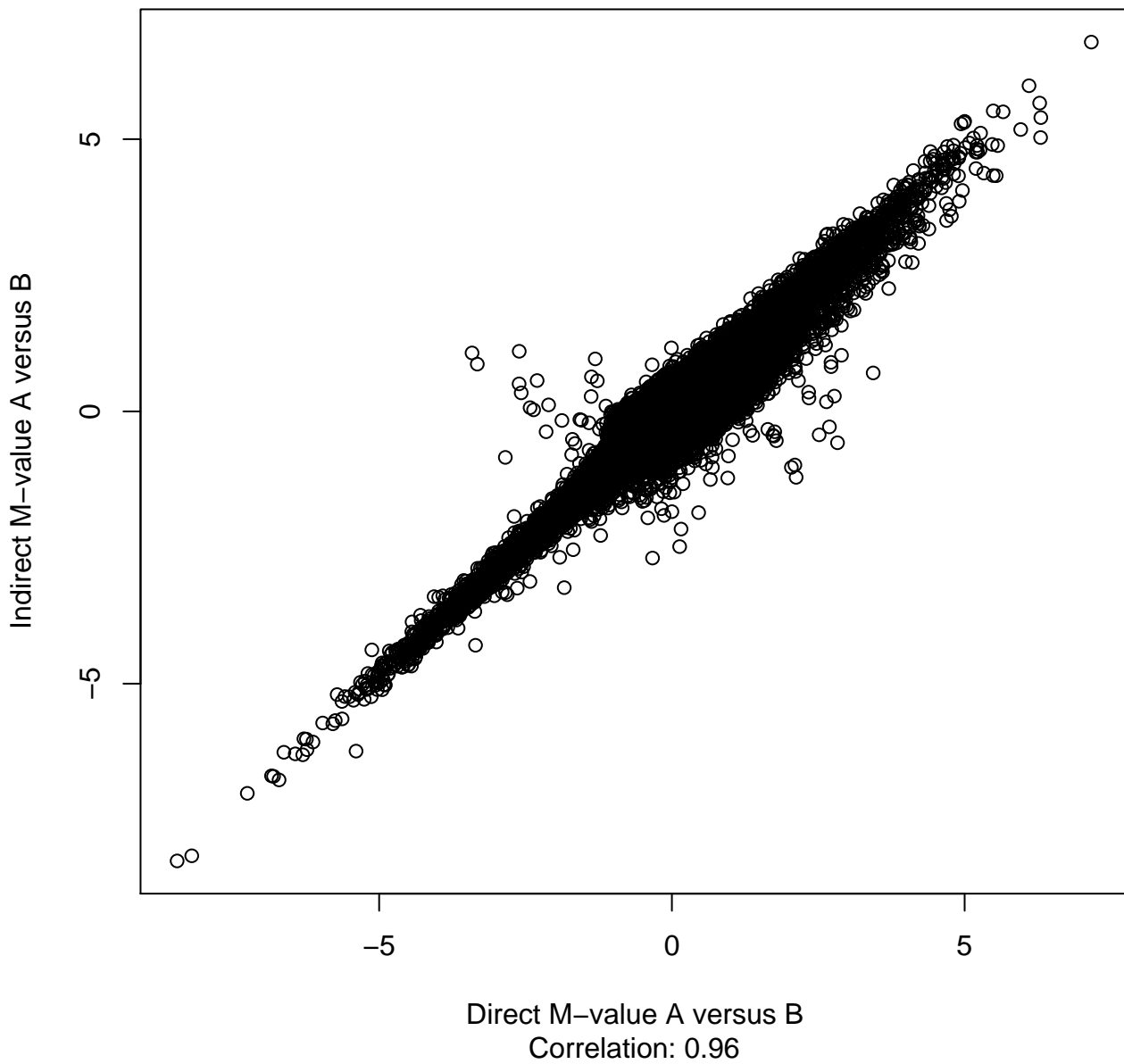

**D2**

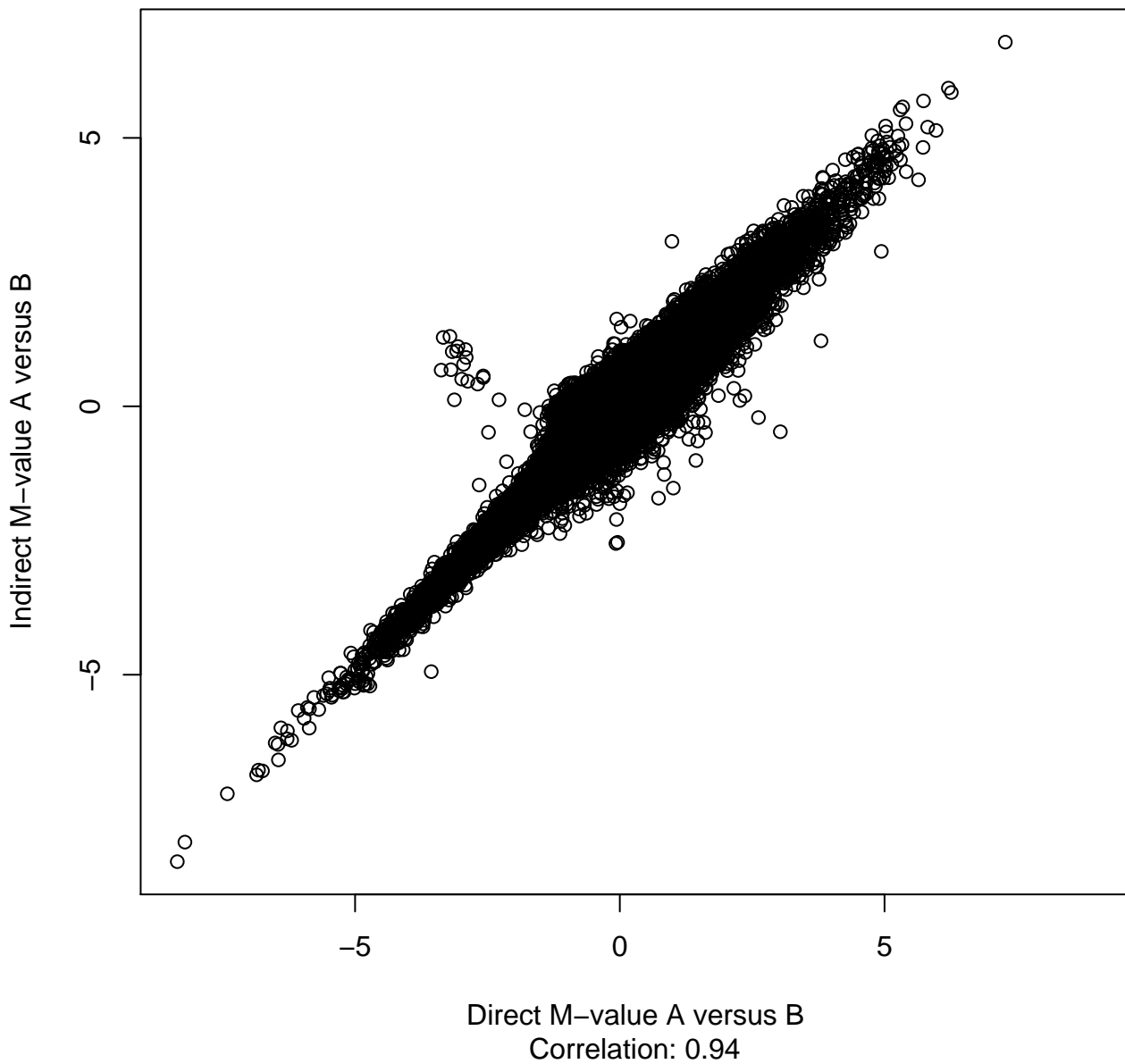

**D3**

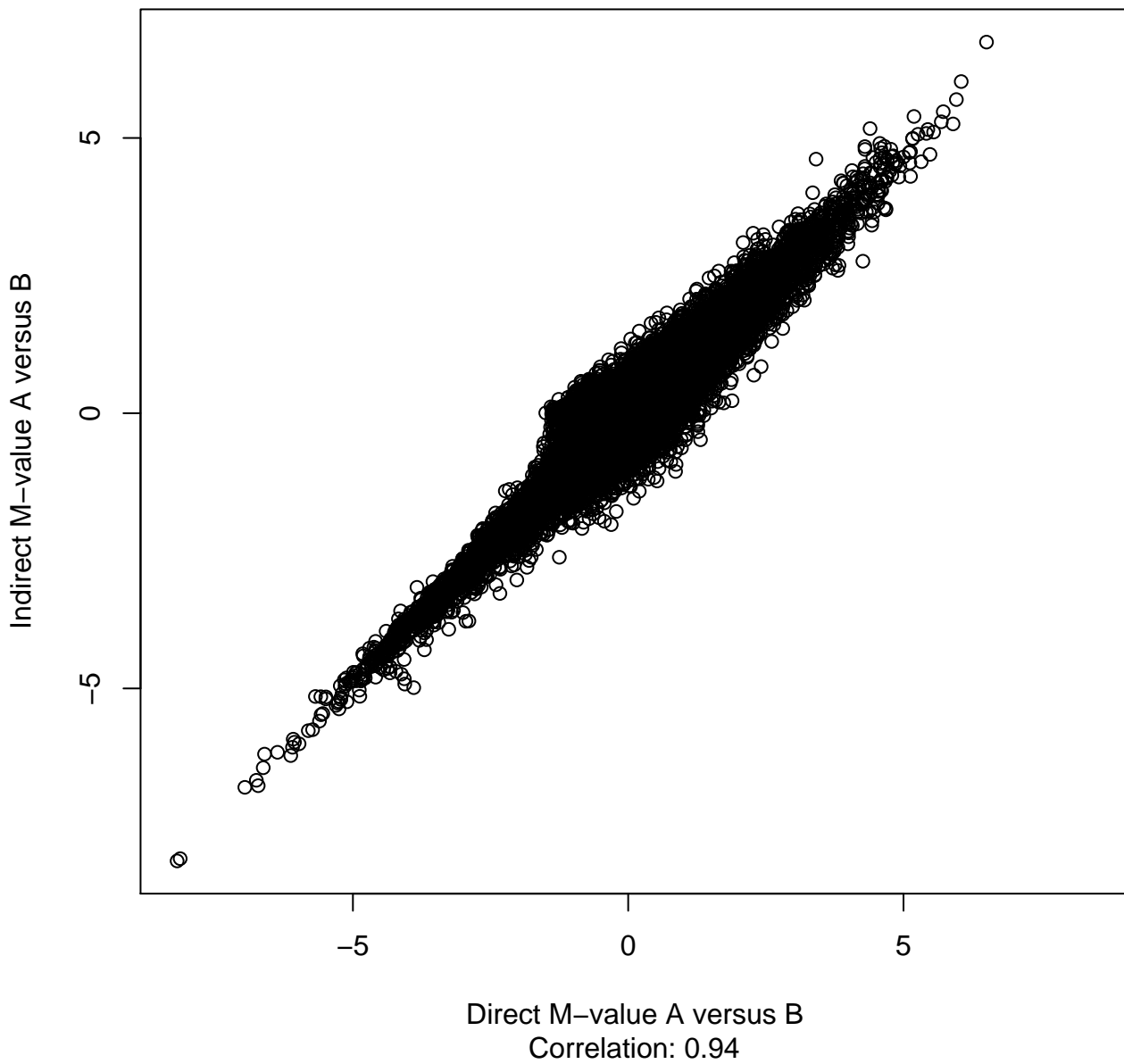

D4

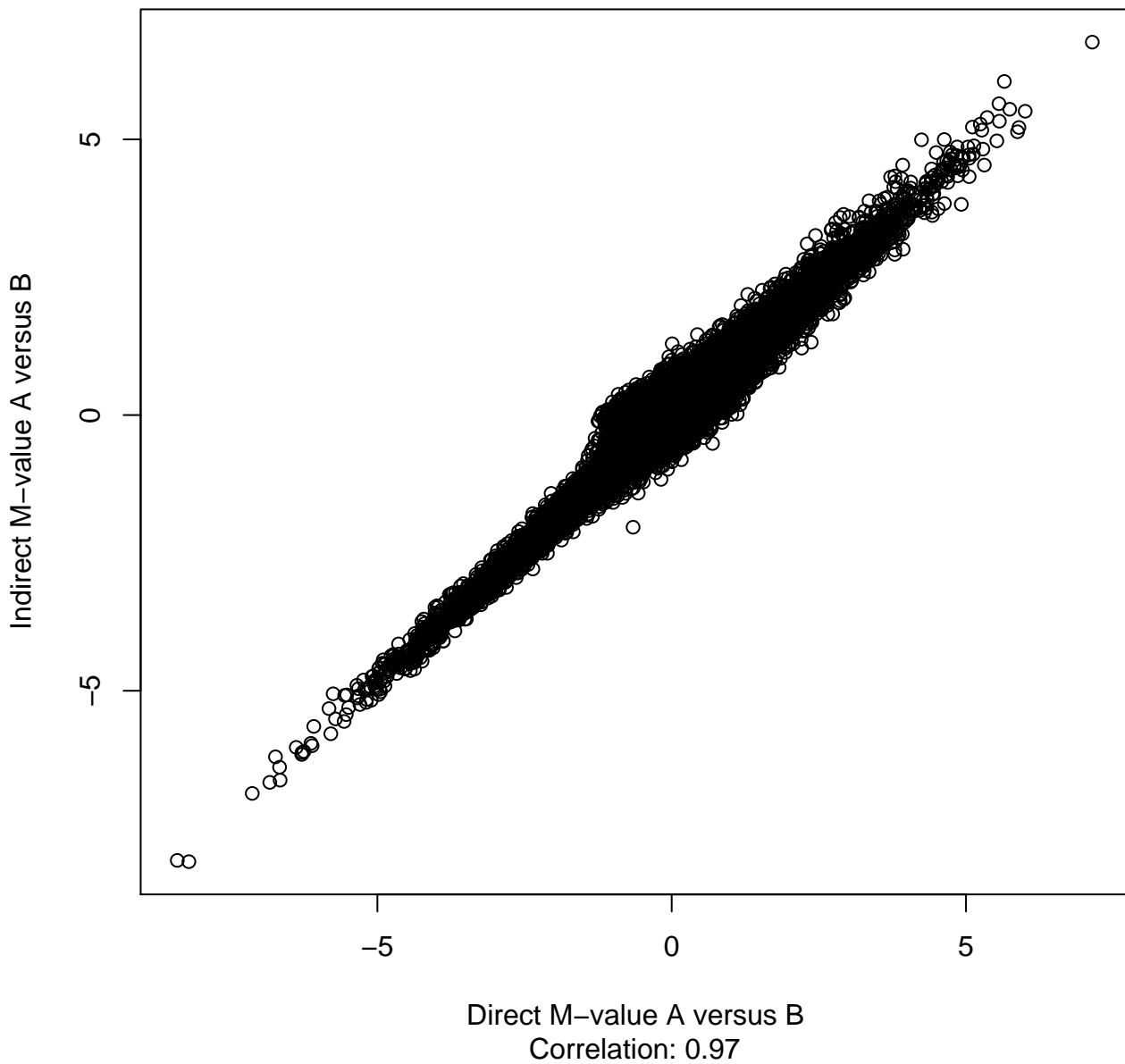

**D5**

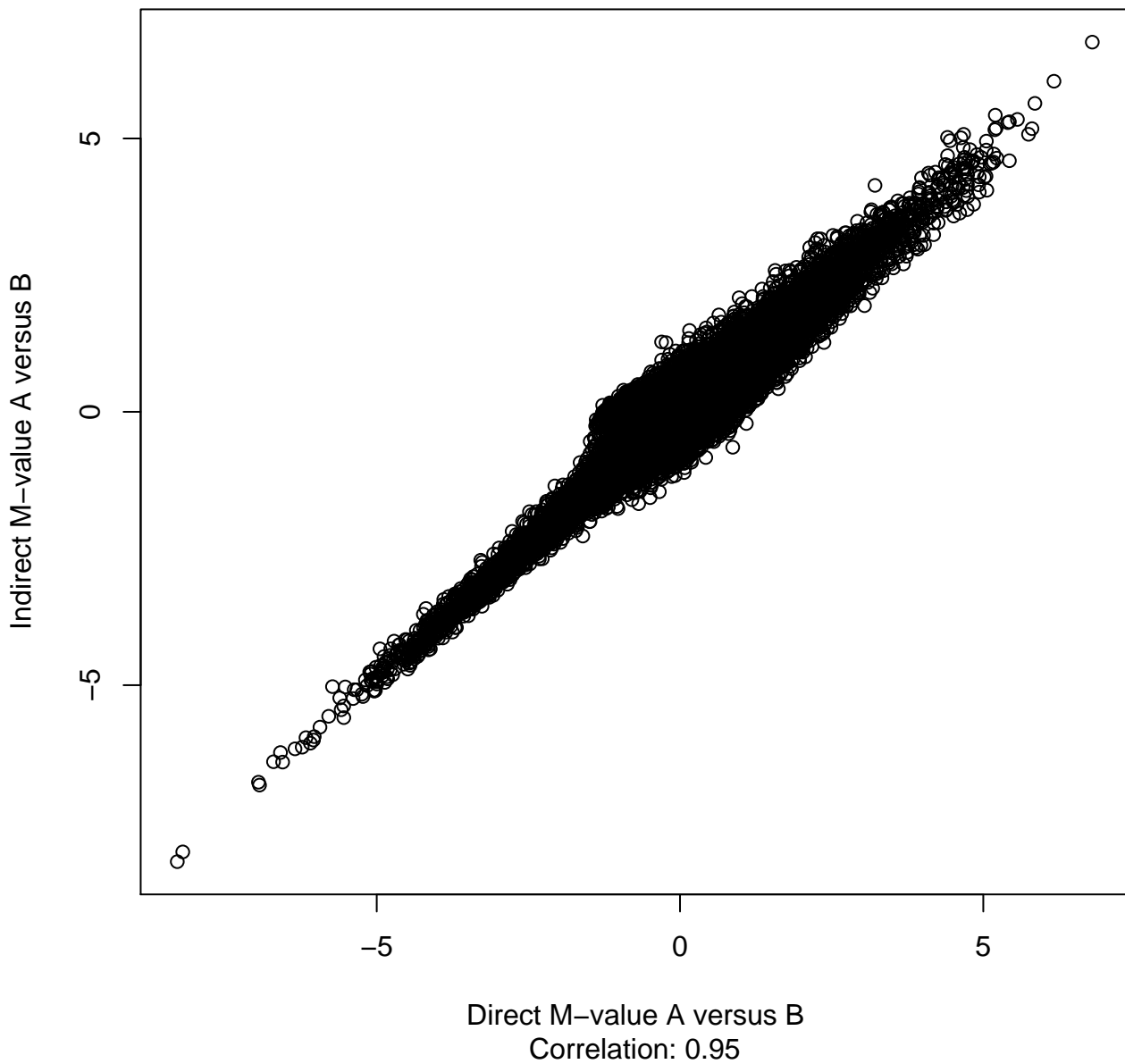

Supplement: Additional file 3 — Correlation real versus virtual hybridizations. Correlation between M-values derived from real hybridizations versus M-values derived from virtual hybridizations for the MAQC dataset. Main title: hybridization number. X-axis: directly measured ratio between sample A and B. Y-axis: mean M-value between 9 virtual comparisons sample A versus sample B, where sample A and B where measured on different arrays. Correlation: Pearson's correlation coefficient. [file 1471-2164-11-112-S3.ZIP › test.pdf]

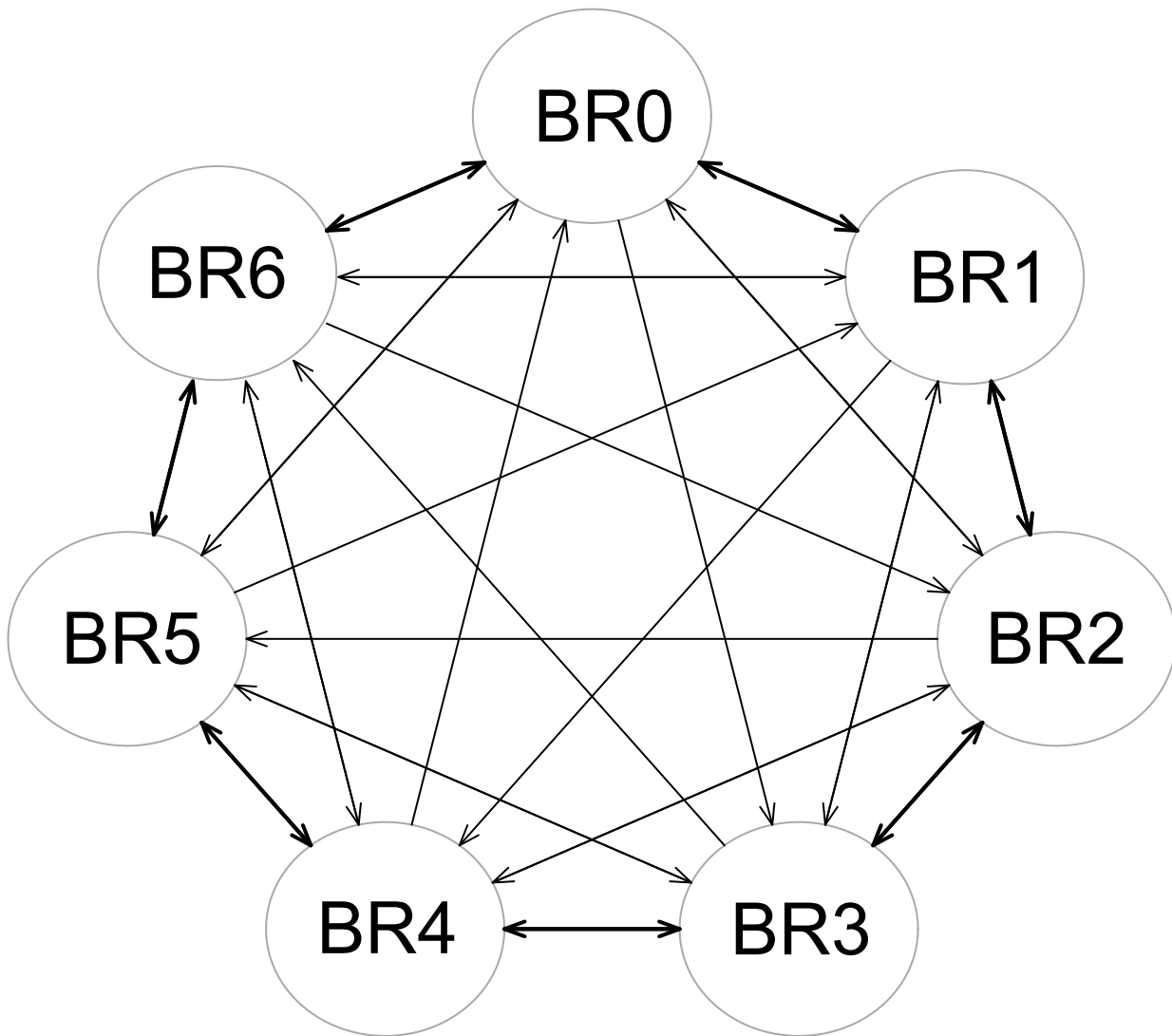

↔ n=4 arrays  
↔ n=2 arrays  
→ n=1 arrays

Supplement: Additional file 6 — Hybdridization setup brain experiment. BR = Braak stage. [file 1471-2164-11-112-S6.PDF]
