# Supplementary material for: Intensity-based analysis of dual-color gene expression data as an alternative to ratio-based analysis to enhance reproducibility
Source: BMC Genomics. 2010 Feb 17;11:112. doi: 10.1186/1471-2164-11-112 (PMC2838842; doi:10.1186/1471-2164-11-112)
Supplement: Additional file 5 — Reproducibility of between-group treatment effects based on ratio and intensity models. Reproducibility of ANOVA-derived treatment effects between group 2 and group 4, and group 1 and group 5, in replicate brain datasets B1 and B2. Reproducibility is enhanced when using intensity-based models instead of ratio-based models. [file 1471-2164-11-112-S5.PDF]

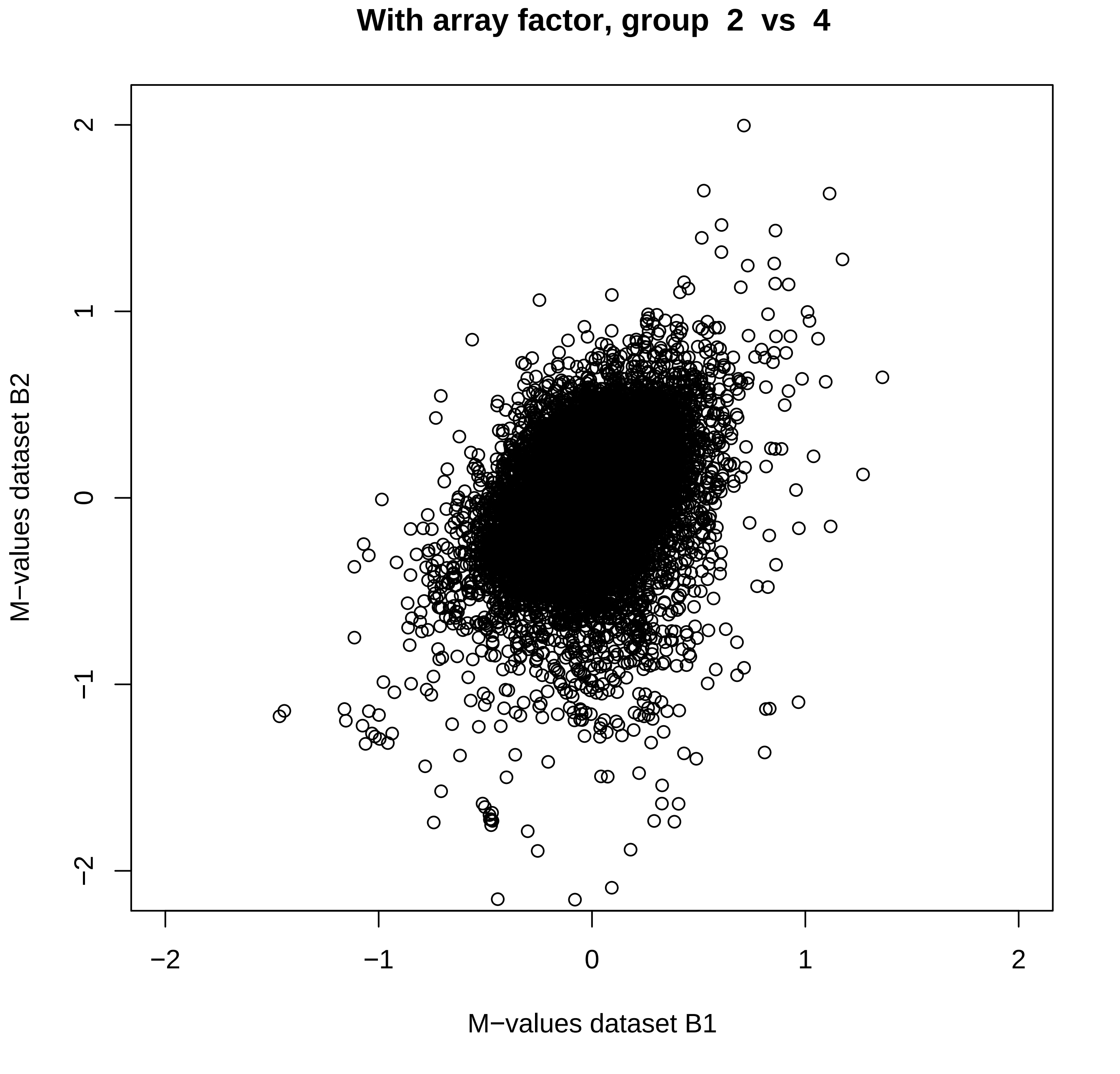
With array factor, group 2 vs 4
M−values dataset B2
M−values dataset B1
−2
−1
0
1
2
−2
−1
0
1
2

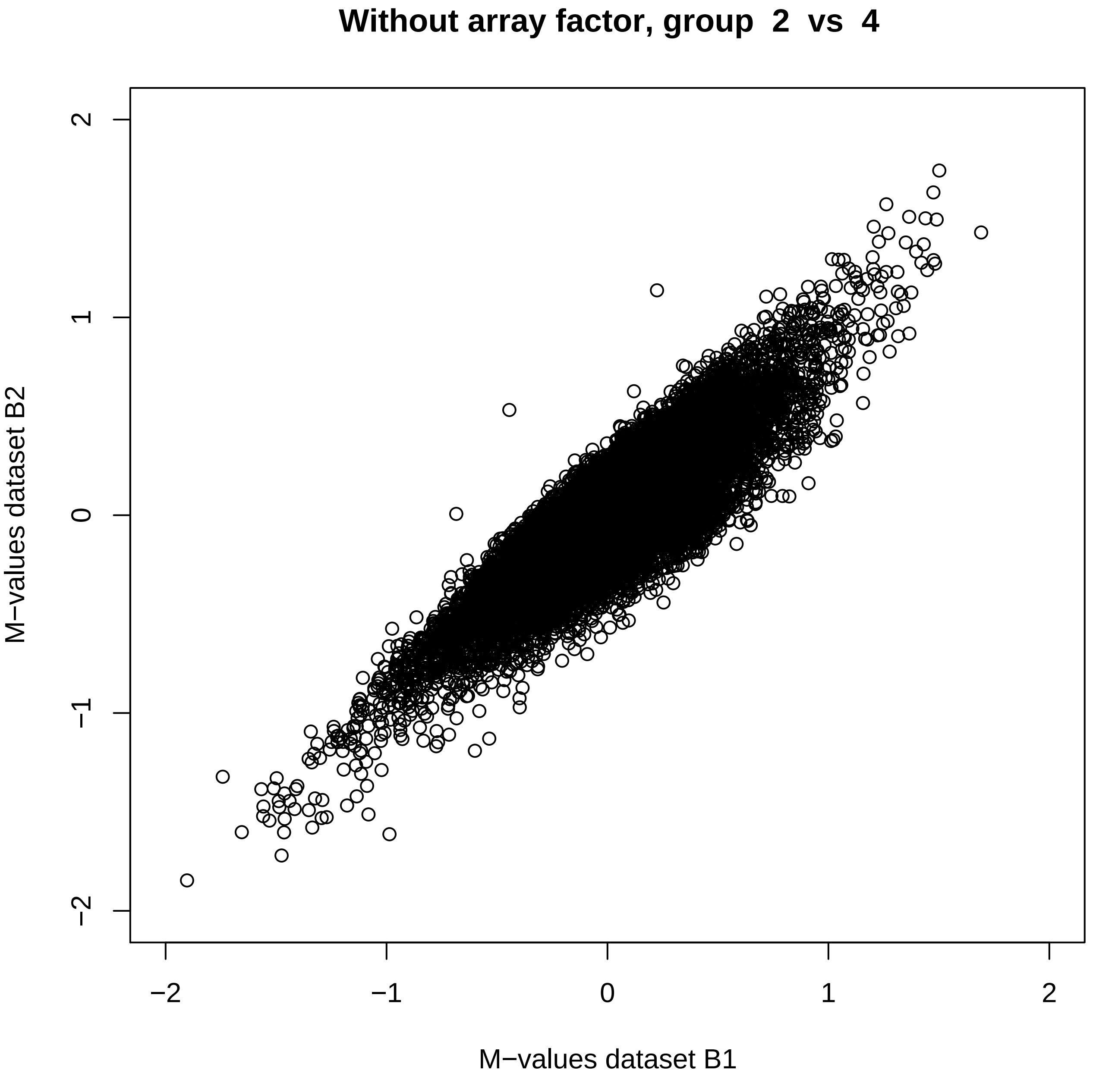

Without array factor, group 2 vs 4
M−values dataset B2
M−values dataset B1
−2
−1
0
1
2

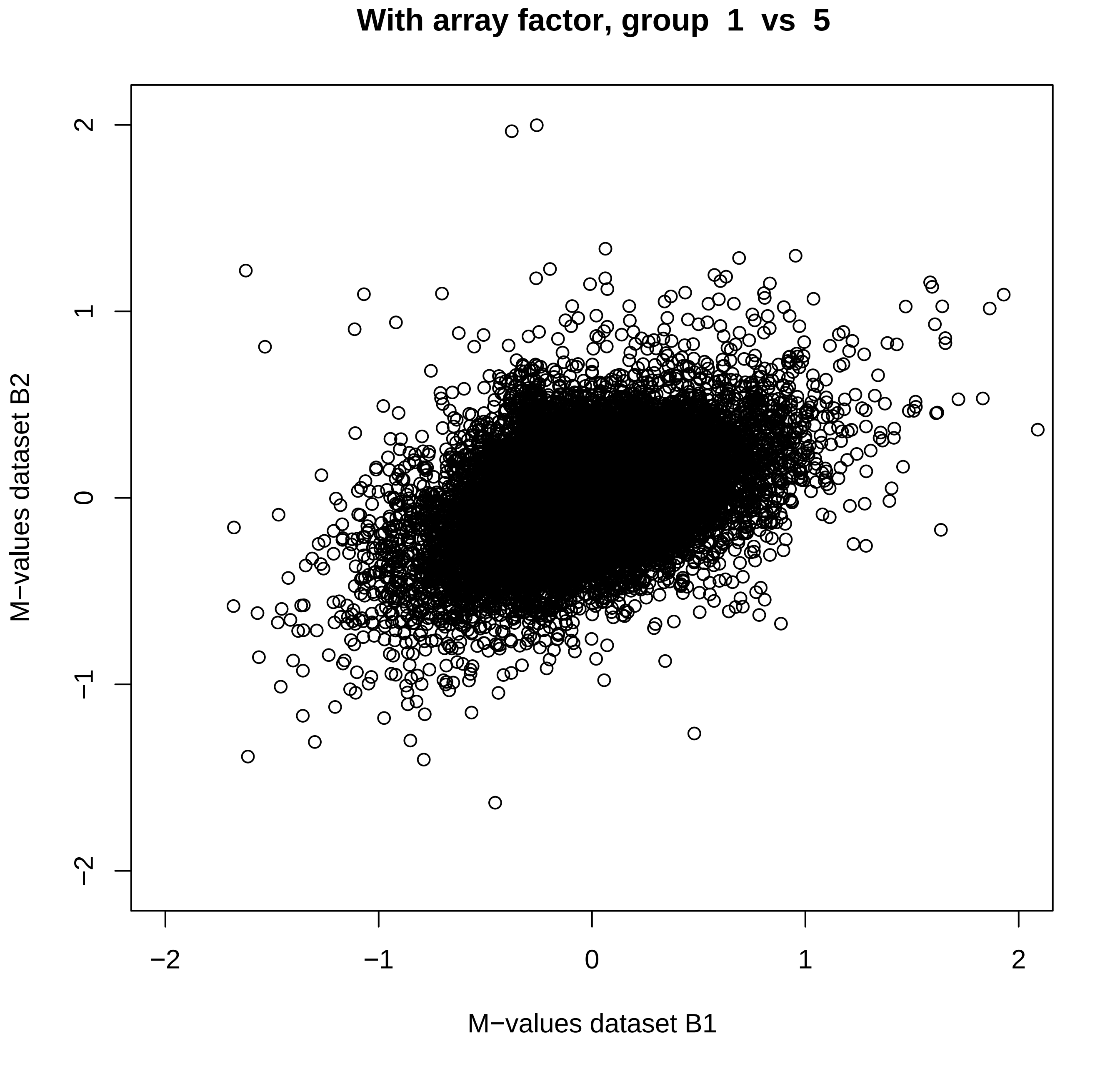

With array factor, group 1 vs 5
M−values dataset B2
M−values dataset B1
2
1
0
−1
−2
−2
−1
0
1
2

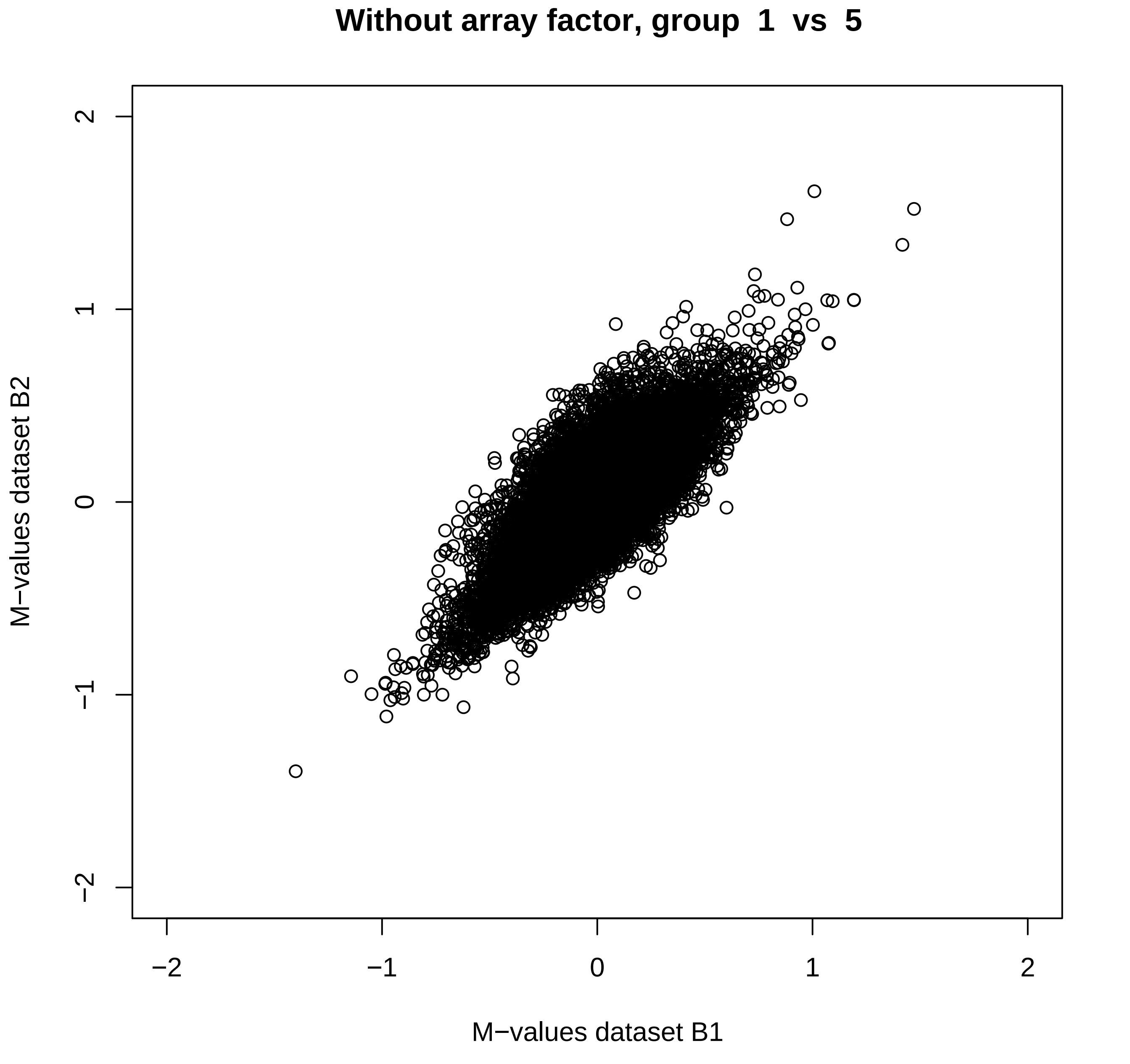

Without array factor, group 1 vs 5
M−values dataset B2
M−values dataset B1
−2
−1
0
1
2
−2
−1
0
1
2
